# Environmentally relevant concentrations of titanium dioxide nanoparticles pose negligible risk to marine microbes†

**DOI:** 10.1039/d0en00883d

**Published:** 2021-04-09

**Authors:** Craig J. Dedman, Aaron M. King, Joseph A. Christie-Oleza, Gemma-Louise Davies

**Affiliations:** School of Life Sciences, Gibbet Hill Campus, University of Warwick Coventry CV4 7AL UK C.Dedman@warwick.ac.uk; Department of Chemistry, University of Warwick Gibbet Hill Coventry CV4 7EQ UK; UCL Department of Chemistry, University College London 20 Gordon Street London WC1H 0AJ UK gemma-louise.davies@ucl.ac.uk; Department of Biology, University of the Balearic Islands Ctra. Valldemossa, km 7.5 CP: 07122 Palma Spain; IMEDEA (CSIC-UIB) CP: 07190 Esporles Spain joseph.christie@uib.eu

## Abstract

Nano-sized titanium dioxide (nTiO_2_) represents the highest produced nanomaterial by mass worldwide and, due to its prevalent industrial and commercial use, it inevitably reaches the natural environment. Previous work has revealed a negative impact of nTiO_2_ upon marine phytoplankton growth, however, studies are typically carried out at concentrations far exceeding those measured and predicted to occur in the environment currently. Here, a series of experiments were carried out to assess the effects of both research-grade nTiO_2_ and nTiO_2_ extracted from consumer products upon the marine dominant cyanobacterium, *Prochlorococcus*, and natural marine communities at environmentally relevant and supra-environmental concentrations (*i.e.*, 1 μg L^−1^ to 100 mg L^−1^). Cell declines observed in *Prochlorococcus* cultures were associated with the extensive aggregation behaviour of nTiO_2_ in saline media and the subsequent entrapment of microbial cells. Hence, higher concentrations of nTiO_2_ particles exerted a stronger decline of cyanobacterial populations. However, within natural oligotrophic seawater, cultures were able to recover over time as the nanoparticles aggregated out of solution after 72 h. Subsequent shotgun proteomic analysis of *Prochlorococcus* cultures exposed to environmentally relevant concentrations confirmed minimal molecular features of toxicity, suggesting that direct physical effects are responsible for short-term microbial population decline. In an additional experiment, the diversity and structure of natural marine microbial communities showed negligible variations when exposed to environmentally relevant nTiO_2_ concentrations (*i.e.*, 25 μg L^−1^). As such, the environmental risk of nTiO_2_ towards marine microbial species appears low, however the potential for adverse effects in hotspots of contamination exists. In future, research must be extended to consider any effect of other components of nano-enabled product formulations upon nanomaterial fate and impact within the natural environment.

## Abstract 2

Exposure of *Prochlorococcus* cultures to research-grade and extracted nano-sized TiO_2_ at environmentally-relevant and supra-environmental concentrations (1 μg L^−1^ to 100 mg L^−1^) results in initial cell decline, followed by full population recovery.

## Graphical abstract

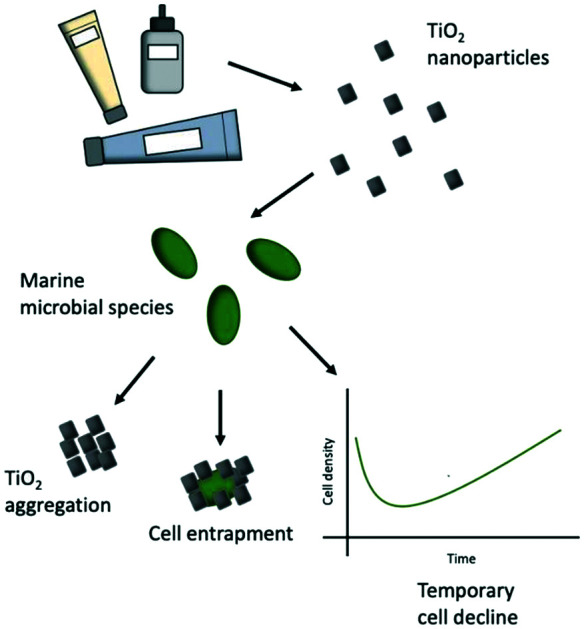

**Environmental significance**

Titanium dioxide (nTiO_2_) represents the highest produced engineered nanomaterial worldwide, yet its impact in the marine environment is poorly understood. Despite 70–80% use in the cosmetics industry, typically only pristine research-grade materials are examined. Herein, the effects of research-grade and nTiO_2_ extracted from common consumer goods upon marine microbial species were investigated. We show cell decline of the cyanobacterium *Prochlorococcus* during short-term exposure (72 h), but ultimate recovery under environmentally relevant conditions after 10 days. Cell decline following nTiO_2_ exposure appear to result from nanoparticle aggregation and entrapment of cyanobacteria, while little molecular features of toxicity are identified. Exposure of natural communities reveals negligible effect of nTiO_2_ on marine microbial community structure. Therefore, the risk of nTiO_2_ exposure in the marine environment appears low.

## 1. Introduction

The fate and effects of engineered nanomaterials (NMs) within the natural environment has become an ecological concern over the past decade.^1–3^ Nano-sized titanium dioxide (nTiO_2_) represents the highest produced NM worldwide, with annual production predicted to reach 2.5 million tonnes by 2025.^4,5^ Due to its prevalent use across a wide range of industries, including plastic production, paints, foods and cosmetics,^5,6^ nTiO_2_ is highly likely to enter the natural environment.^7^ The cosmetics industry dominates the use of nTiO_2_, accounting for approximately 70–80% of nTiO_2_ use.^6^ Typically product formulations are made up of 2–14 wt% nTiO_2_,^8,9^ however, the exact physicochemical properties of NMs used vary greatly. In fact, it is estimated that the chemical composition of NMs utilised for over 50% of commercial products is not widely publicised.^4^

Domestic use of consumer products such as sunscreen and toothpaste is predicted to release considerable volumes of TiO_2_ into the aquatic environment.^6,10^ Extraction of metal oxide NMs from wastewater treatment works has been recorded at an efficiency of approximately 95%,^11^ resulting in the entry of approximately 5% of metal oxide NMs into the aquatic environment *via* wastewater effluent. Given that 4100 tonnes of TiO_2_ is predicted to enter US wastewaters as a result of toothpaste use every year,^6^ we can estimate that up to 205 tonnes will enter wastewater effluent and be transported to natural systems, where their ultimate fate remains unknown.

In recent years, increasing efforts have been made to uncover the environmental concentrations of engineered NMs. Difficulties in sampling techniques and varying effectiveness of methods for characterising nano-pollutants, particularly at the low concentrations predicted in the environment, has led to a lack of environmental data.^12,13^ ‘Environmental Fate Models’ represent a powerful tool for this purpose,^14^ and surface water concentrations of nTiO_2_ are estimated in the range of 0.021–10.000 μg L^−1^.^15^ However, more recently, environmental sampling has revealed concentrations of up to 40 μg L^−1^ close to major transport infrastructure and locations heavily impacted by tourism.^16,17^ For example, during the peak tourist season, TiO_2_ derived from sunscreen use has been measured in the range of 7–40 μg L^−1^ in surface waters off the Mallorcan coast.^17^ As such, it appears that the environmental risk from nano-sized pollutants is likely focused within localised areas of contamination which may vary temporally, where particle-specific properties govern their subsequent fate and transport.^18^

The microbial community plays a fundamental role in the functioning of the marine ecosystem, contributing approximately 50% of global primary productivity and influencing major climatic and biogeochemical cycles.^19,20^ Hence, understanding the potential effects of contaminants upon marine microbes is key to evaluating their likely ecosystem-wide impact. Previous research has revealed a toxic effect of nTiO_2_ exposure upon marine microbial species,^6,21–30^ although studies where little or no adverse effect is recorded also exist.^31,32^ The primary effect of exposure appears to be growth inhibition, however EC_50_ values are typically recorded in the mg L^−1^ range, far greater than those measured in the environment (up to 40 μg L^−1^).^23–25^ In addition to growth inhibition, negative effects such as the induction of oxidative stress pathways, physical damage to cells, and entrapment of cells within aggregates of NMs have been recorded following exposure, however results vary greatly.^23–25,32,33^ Indeed, physical interaction between phytoplankton and aggregates of nTiO_2_ appears widespread in laboratory exposure^34–37^ and may represent a possible pathway for phototrophic removal from epipelagic layers^35^ or transport of NMs to higher trophic levels.

Much of the toxicological work of nTiO_2_ has so far been carried out using standardised research-grade NMs, which are not usually surface modified. More recently, studies have begun to emerge focussing upon the toxicity of surface functionalised nTiO_2_ (*e.g.* sunscreens which often possess surfactant/polymer surface modifications) upon marine phytoplankton.^6,27^ In these studies, adverse effects were observed in phytoplankton exposed to sunscreens containing nTiO_2_, associated with increased production of reactive oxygen species (ROS), membrane damage and possible genotoxicity.^27^ However, biostimulating effects of nTiO_2_-containing sunscreens have also been recorded, attributed to other organic components of the sunscreen formulation.^8^ Interestingly, nTiO_2_ derived from commercial products have been reported to result in greater growth inhibition than research-grade nanoparticles.^6^ It should be noted, though, that much of the work investigating metal oxide NMs are carried out using exposure concentrations in the mg L^−1^ range (*i.e.* 1–30 mg L^−1^),^6,21–30^ far exceeding those predicted and measured in the environment (0.021–40 μg L^−1^).^6,8,15,17^ Increased research is required to examine the end-products of consumer goods which may enter the aquatic environment.^8^^9^ Given the great variation in physicochemical properties between specific NMs, and hence varied fate in aquatic media, it is important that representative NMs derived from consumer goods are utilised alongside research-grade materials during experimentation.^9,38^ To provide a greater understanding of the environmental impact of nTiO_2_, research must also be directed to simulate environmental conditions as effectively as possible.

Since much of the previous research in this field has focussed upon phytoplankton species such as diatoms and green algae, comparatively little evidence for the effects of NM exposure upon photosynthetic cyanobacteria exist. Marine cyanobacteria, mainly *Prochlorococcus* and *Synechococcus* species, are the most abundant photosynthetic organisms on earth and major contributors to global primary productivity.^39,40^ Furthermore, among phytoplankton taxa, cyanobacteria appear particularly sensitive to nano-pollutants *e.g.* silver nanoparticles.^41^ Herein, we aimed to provide a broad-spectrum analysis of both commercially available research-grade (non-surface modified) nTiO_2_, as well as nTiO_2_ extracted from common consumer products, and examined their impact upon marine phytoplankton at both the organism- and community-level. Short-term (72 h) and medium-term (10 d) toxicity of nTiO_2_ was examined using the ecologically significant cyanobacterium *Prochlorococcus* sp. MED4 under environmentally relevant conditions (*i.e.*, at ambient cell densities (10^4^–10^5^ cells per mL),^42,43^ in oligotrophic natural seawater). The behaviour of nTiO_2_ in seawater and its interaction with cyanobacteria was investigated through the use of dynamic light scattering (DLS), flow cytometry and fluorescent microscopy. Molecular features of toxicity were assessed by shotgun proteomic analysis and appeared negligible. Finally, an additional experiment was conducted using natural coastal seawater to characterise the whole community response towards consumer nTiO_2_ exposure. Amplicon sequencing of the 16S rRNA and 18S rRNA genes revealed little impact of extracted nTiO_2_ derived from sunscreen upon natural marine microbial community structure at environmental concentrations. This multi-OMIC study provides a comprehensive assessment of the differing effects that result from exposure to various types of nTiO_2_, representative of materials likely to enter the marine environment, thus facilitating effective evaluation of their likely interaction with marine microbial species and overall environmental risk.

## 2. Methods

### 2.1 Materials

Research-grade nTiO_2_ used during experimentation was purchased from Sigma Aldrich (21 nm (19.9 ± 6.6 nm, TEM)). Three consumer products; Skinceuticals™ sunscreen (S1), Boots Soltan™ sunscreen (S2), and The Body Shop™ liquid foundation (P1) were selected based on nTiO_2_ being listed as an ingredient and purchased from a high street retailer. Natural seawater (NSW) obtained from Station L4 (Plymouth, UK, 50°15.0′N; 4°13.0′W) was routinely used during experimental work. Prior to use, NSW was autoclaved and filtered (0.22 μm, polyethersulfone membrane Corning®). ‘Neat’ sunscreen stocks were prepared by diluting the cream directly in NSW. For all experimental work, nTiO_2_ stocks were prepared in NSW and sonicated for 15–30 min (Branson 1210 Sonicator, 40 kHz) prior to addition to experimental media to avoid extensive aggregation. Glassware was acid-washed before use. Axenic *Prochlorococcus* sp. strain MED4 was routinely grown using Pro99 media^44^ and maintained at 23 °C before use in experimentation.

### 2.2 Extraction of nTiO_2_ from consumer products

Methods adapted from those described by Galletti *et al.* (2016) were utilised to extract nTiO_2_ from consumer goods.^6^ Briefly, 1.5–2.5 g of product was soaked in 20 mL hexane for 2 h. Suspensions were then shaken manually and centrifuged at 4400 rpm for 5 min. The supernatant was subsequently discarded, and 20 mL ethanol was added and shaken manually. The solution was then centrifuged at 4400 rpm for 5 min and the resultant supernatant was discarded. Following this, the remaining product was washed in Milli-Q ultrapure H_2_O (0.22 μm filter operated at 18.2 MΩ at 298 K) three times using centrifugation; first centrifuging at 11 000 rpm for 5 min, extending this to 10 min for the final two washes. Extracted pellets were subsequently dried in a 60 °C oven to form a powder. Samples were stored in darkness prior to characterisation and use in experiments with marine phytoplankton.

### 2.3 Characterisation of materials

Transmission electron microscopy (TEM) was utilised to determine primary nanoparticle size. A JEOL 2100 TEM, 200 kV, LaB_6_ instrument operated with a beam current of ∼115 mA was used to obtain images using a Gatan Orius 11-megapixel camera. Samples were prepared by deposition and drying of nanoparticle samples (10 μL aqueous suspension) onto formvar-coated 300 mesh copper TEM grids (EM Resolutions). Diameters were measured using ImageJ version 3.2; average values were calculated by measuring the diameter of >100 particles with errors represented as standard deviations. Energy-dispersive X-ray spectroscopy (EDS) was collected using an Oxford Instruments X-Max 80T detector. Additionally, samples were ground to fine powders before powder X-ray diffraction (P-XRD) was performed using a STOE Stadi-P diffractometer with a molybdenum X-ray source (operated at 50 kV and 30 mA), *λ* = 0.7093 Å. The 2*θ* scan range was 2–40.115° at a step size of 0.495° and 5 seconds per step. Samples were prepared using STOE zero scattering foils before being inserted into the transmission sample holder.

### 2.4 Short-term (72 h) consumer nTiO_2_ exposure

*Prochlorococcus* MED4 was inoculated into oligotrophic NSW at ambient cell densities (∼10^4^ cells per mL) and incubated 72 h for pre-adaptation to the oligotrophic conditions prior to experimentation (*i.e.*, 23 °C at constant 10 μmol photons m^−2^ s^−1^ light intensity, using a Lifelite™ full spectrum bulb with UV, and with shaking at 100 rpm). The light intensity used is optimal for cultured *Prochlorococcus* sp. MED4 and ensured cyanobacteria were not affected by light stress during experiments. 30 mL of pre-adapted *Prochlorococcus* culture was aliquoted into 50 mL tissue culture flasks and spiked with nTiO_2_ stocks to make up test concentrations of 0, 5, 50 and 500 μg L^−1^, all in triplicate. Four nTiO_2_ treatments were investigated: nTiO_2_ nanopowder (Sigma Aldrich), nTiO_2_ derived from sunscreen S1 and S2, and nTiO_2_ derived from liquid foundation P1. Additionally, a ‘neat’ sunscreen treatment was tested, where 0.1 g of sunscreen S2 was immersed in NSW and mixed *via* manual shaking and 15 min sonication. Cultures were subsequently spiked with a defined volume of the sunscreen suspension to make up equivalent test concentrations based on nTiO_2_ making up ∼10 wt% of the sunscreen formulation.^8,9^ After the addition of each treatment, cultures were further incubated under the conditions described above and monitored by flow cytometry using a Becton Dickinson Fortessa flow cytometer at time points 0, 24, 48 and 72 h. Cell densities were calculated in respect to reference beads (2.2 μm high intensity fluorescent Nile Red particles (Spherotech FH-2056-2)), added to samples at a defined concentration. For additional details of flow cytometric analyses used throughout experimentation see Section S2, ESI.†

### 2.5 Medium-term (10 d) nTiO_2_ exposure

To investigate the medium-term effects of nTiO_2_ exposure upon *Prochlorococcus* MED4, research-grade nTiO_2_ (Sigma Aldrich, 19.9 ± 6.6 nm) was utilised for experimentation, based on results of earlier experiments described in section 2.4. Two culture conditions were tested: i) cell-dense cultures (∼10^6–7^ cells per mL) in nutrient-rich Pro99 media, and ii) cultures grown to ambient cell densities (∼10^4–5^ cells per mL) in oligotrophic NSW, representing environmental conditions. Cultures were set up as described in section 2.4. Culture flasks were subsequently spiked with a nTiO_2_ stock achieving final concentrations in the μg L^−1^ (1, 10 and 100 μg L^−1^) and mg L^−1^ range (1, 10 and 100 mg L^−1^), all in triplicate; representing environmental and supra-environmental concentrations respectively. Cell counts were monitored at 0, 24, 48, 72, 192 and 240 h by flow cytometry as previous and compared to that of an untreated control. In addition to monitoring of cell density, flow cytometric analysis was utilised to infer the behaviour of nTiO_2_ within test media and their interaction with cyanobacterial cells (see Section S2.1,† for further information).

### 2.6 Imaging of nTiO_2_-cyanobacterial aggregates by fluorescent microscopy

To investigate the hetero-aggregation between nTiO_2_ and *Prochlorococcus*, a 200 μL sample was collected from the bottom of culture flasks from one replicate of each treated group during medium-term experiments (section 2.5). This sub-sample was stained with 1X SYBR Gold nuclear stain (ThermoFisher) and imaging was carried out at 40× magnification using a Nikon widefield fluorescence microscope under brightfield and GFP fluorescence. Images were captured from both channels and subsequently merged to assess the presence and extent of aggregation between cyanobacterial cells and nTiO_2_. Controls containing nTiO_2_ only (100 mg L^−1^) were included to prove the nanoparticles did not get stained by the dye. In addition, NSW samples and untreated *Prochlorococcus* culture in the absence of nTiO_2_ were imaged to confirm aggregates were indeed nTiO_2_ rather than other particulate material.

### 2.7 Examining the behaviour of nTiO_2_ within natural seawater by dynamic light scattering

The aggregation behaviour of nTiO_2_ (Sigma Aldrich, 19.9 ± 6.6 nm) within NSW was assessed by *z*-average size (d.nm) over a period of 336 h (14 d). Here, hydrodynamic particle size measurements were determined by dynamic light scattering (DLS) using a Malvern Zetasizer Nano ZS instrument, equipped with a 4 mW He–Ne 633 nm laser module. A stock of nTiO_2_ was sonicated for 15–30 min prior to addition to NSW. Concentrations of 1 mg L^−1^ and 100 mg L^−1^ were utilised due to limitations of the DLS at lower concentrations, hence hindering the ability to assess nTiO_2_ aggregation at the environmentally relevant concentrations (*i.e.*, in the μg L^−1^ range) used in this study during toxicity testing. nTiO_2_ suspensions were made up in 20 mL autoclaved and filtered (0.22 μm) NSW in 50 mL tissue culture flasks and placed on an orbital shaker (100 rpm) to simulate natural movement of water. DLS measurements were carried out upon a 200 μL sub-sample collected from the mid-point of flasks at set timepoints (0, 1, 2, 4, 24, 48, 72, 168, 240, 336 h). For each sample the average was taken from 3 measurements made up of 11 sampling runs lasting 10 s each.

### 2.8 Shotgun proteomic analysis

To ensure sufficient biological material was obtained for proteomic analyses, cell-dense *Prochlorococcus* MED4 cultures were grown in Pro99 media. Following 72 h preadaptation to experimental conditions, described above (section 2.4), triplicate cultures were spiked with research-grade nTiO_2_ stock (Sigma Aldrich, 19.9 ± 6.6 nm) to achieve a test concentration of 100 μg L^−1^. Untreated cultures were also prepared as controls. Following the addition of nTiO_2_, cultures were incubated 24 h, after which samples were immediately centrifuged for 10 min at 4 °C at 4000 × *g* and pellets were immediately frozen in dry ice until further processing. The supernatants containing the extracellular proteome were filtered (0.22 μm) and stored at −20 °C. Supernatants were thawed at room temperature and underwent trichloroacetic acid (TCA) protein precipitation as previously described.^45^ Subsequently, cell and extracellular proteome pellets were resuspended in 1x LDS buffer (ThermoFisher) containing 1% beta-mercaptoethanol and run on a NuPage 4–12% Bis-Tris precast polyacrylamide gels as previously done.^46^ In-gel trypsin digestion and peptide recovery was performed^47^ followed by a nanoLC-ESI-MS/MS analysis using an UltiMate 3000 RSLCnano System coupled to an Orbitrap Fusion (Thermo Scientific) using conditions and settings as previously described.^48^ RAW mass spectral files were processed using MaxQuant version 1.5.5.1 (ref. 49) for peptide identification and protein label-free quantification using the *Prochlorococcus* sp. MED4 UniProt coding domain sequences (downloaded on 16/01/2018). For further details of the laboratory and bioinformatics protocols used for proteomics analysis see Section S4, ESI.†

### 2.9 16S rRNA/18S rRNA amplicon sequencing

A site for experimental work was selected in the Balearic Islands, Spain (39.493868, 2.739820). Field experiments were carried out during April 2018. Here, coastal seawater (NSW) containing its natural microbial community was collected at a depth of approximately 1 m, representing those microorganisms most likely to interact with nTiO_2_ derived from sunscreen in the coastal system. Subsequently, 500 mL was transferred to pre-washed 1 L Nalgene plastic bottles, leaving sufficient volume empty for air exchange. Microbial communities were exposed to one of three treatments: 1) untreated control, where no nTiO_2_ was added; 2) nTiO_2_ extracted from sunscreen S2; 3) ‘Neat’ sunscreen S2 dispersed in NSW. Extracted nTiO_2_ from S2 were obtained as described above (section 2.2) and added at a final concentration of 25 μg L^−1^. ‘Neat’ sunscreen stock was made up in NSW and a defined volume was added to achieve ∼25 μg L^−1^ of nTiO_2_, assuming that nTiO_2_ typically makes up ∼10 wt% of such products.^8,9^ Bottles were mixed by inversion three times and incubated over two days in an outdoor water container to provide temperature stability while being exposed to natural sunlight to best replicate natural conditions. Bottle caps were loosened to ensure sufficient gas exchange and bottles were shaken manually (15–30 s) at regular intervals throughout the experiment to ensure water was well mixed. Following exposure, microbial cells were collected by filtering the 500 mL through a 0.22 μm filter (Millipore). Filters were transferred to a 2 mL Eppendorf containing lysis buffer (Qiagen) and stored at −20 °C. DNA extraction was carried out using the DNeasy Power Biofilm extraction kit (Qiagen) according to the manufacturer's instructions, including a bead beating step as previously described.^50^ DNA quantification was obtained by Qubit® HS DNA kit (Life Technologies Corporation). Extracted DNA samples were stored at −20 °C. Prokaryotic and eukaryotic community analysis was performed by amplicon sequencing using the 515F-Y and 926R primers to amplify the 16S rRNA v4–5 regions, and V8F and 1510R primers to amplify the 18S rRNA v8–9 regions, respectively.^51,52^ PCR products were purified, indexed, normalized and analysed by 2 × 300 bp paired-end sequencing using the MiSeq system with v3 reagent kit (Illumina) as described in Wright *et al.* 2019.^50^ Raw sequencing data was analysed using the DADA2 bioinformatic pipeline based on its enhanced taxonomic resolution compared to alternative methods.^50,53–55^ For additional details of the amplicon sequencing methods and data analysis used, see Section S5.1, ESI.†

### 2.10 Statistical analysis

To identify significant alterations in cell density recorded during toxicity tests with *Prochlorococcus* MED4 (sections 2.4 and 2.5) two-way *t*-tests were carried out between untreated controls and cultures exposed to various nTiO_2_ treatments at each timepoint. Downstream statistical analysis of shotgun proteomics data (section 2.8) was carried out using Perseus version 1.5.5.3,^56^ following the pipeline described previously.^57^ The mass spectrometry data have been deposited to the ProteomeXchange Consortium (http://proteomecentral.proteomexchange.org) *via* the PRIDE partner repository^58^ with the dataset identifier PXD024726. For amplicon sequencing data collected as described in section 2.9, taxonomically assigned data in the form of amplicon sequencing variants (ASVs) were analysed using MicrobiomeAnalyst software.^59,60^ Briefly, following normalisation by total sum scaling, principle coordinates analysis (PCA) based on Bray–Curtis dissimilarity, in conjunction with permutational multivariate analysis of variance (PERMANOVA) was utilised to assess significant alterations in community composition between treatments at the individual ASV level. Subsequently, two-way *t*-tests were utilised to identify significant variations in relative abundance of various taxonomic groups between control and treated samples. Sequence files have been deposited in the NCBI Short Read Archive (SRA) database under Bioproject: PRJNA690209.

## 3. Results and discussion

### 3.1 Characterisation of research-grade and consumer nTiO_2_

**Fig. 1:**
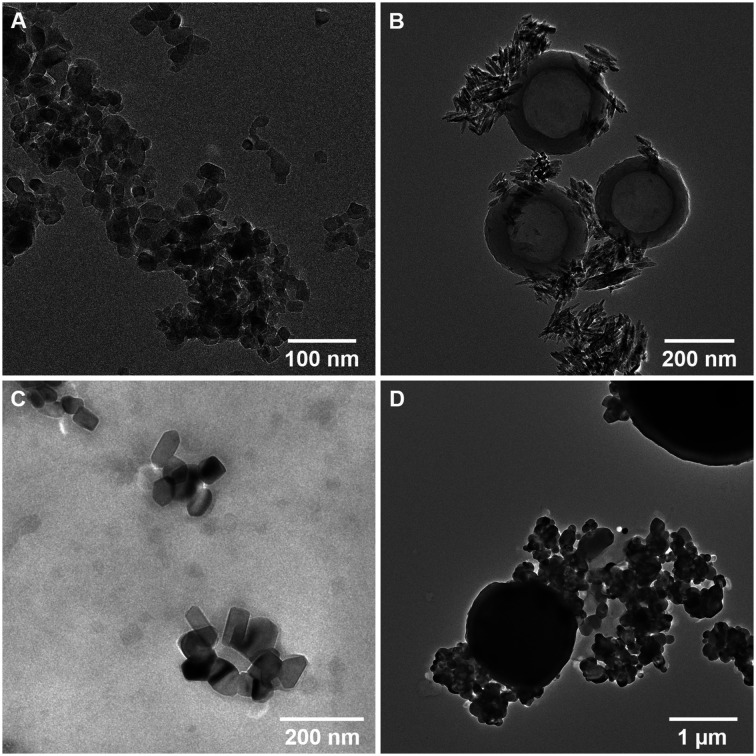
TEM images of nTiO_2_ utilised in experimental work; A – research-grade nTiO_2_ purchased from Sigma Aldrich; B – nTiO_2_ extracted from Skinceuticals™ sunscreen (S1); C – nTiO_2_ extracted from Boots Soltan™ sunscreen (S2); D – nTiO_2_ extracted from The Body Shop™ liquid foundation (P1).

nTiO_2_ was initially extracted from commercial sunscreen and cosmetics products, S1, S2, and P1, as detailed in the Methods section, and characterised. The extraction method utilised to extract particles from the three product formulations was largely effective, yielding a visible powder that could be dried and stored for experimental use. Characterisation of research-grade and extracted materials was carried out using a combination of TEM and EDS mapping, revealing primary particle size, morphology and elemental composition (Table 1 and Fig. 1).

**Table 1:** Summary of material characteristics as determined by TEM, EDS mapping and P-XRD

nTiO_2_ source	Primary particle characteristics				Secondary particle characteristics		
	Size (nm) (TEM)	Elemental composition	Phase	Morphology (TEM)	Size (nm) (TEM)	Elemental composition	Morphology (TEM)
Sigma Aldrich	19.9 ± 6.6	TiO_2_	Anatase and rutile	Mixed cuboid and spherical	n/a	n/a	n/a
Sunscreen S1	50.0 ± 32.9	TiO_2_	Rutile	Needle shaped	294.3 ± 37.5	Carbon-based	Spherical
Sunscreen S2	64.6 ± 26.4	TiO_2_	Rutile	Cuboid	n/a	n/a	n/a
Product P1	158.1 ± 68.7	TiO_2_	Anatase	Cuboid	2705.8 ± 1333.8	SiO_2_	Spherical

Research-grade nTiO_2_ (Sigma Aldrich) possessed an average particle size of 19.9 ± 6.6 nm, close to the manufacturer's advertised size (21 nm), whilst materials extracted from consumer products ranged in average primary particle size 50.0 to 158.1 nm. Interestingly, all primary particles extracted from commercially available products had large standard deviations in sizes, indicating a wide size range in particle populations (Table 1). Typically, samples appeared as small aggregates, a common feature often observed as a result of drying of the samples onto TEM grids (Fig. 1). EDS mapping confirmed that all primary particles were entirely composed of Ti and O (Table 1 and Fig. S1†). P-XRD was carried out on all samples (Fig. S2†). This showed research-grade nTiO_2_ to have a mixture of anatase and rutile phases, with peaks corresponding with the JCPDS patterns 21-1272 (ref. 61) and 21-1276 (ref. 62) for the tetragonal structure of anatase and rutile TiO_2_, respectively. The sunscreens S1 and S2 samples appeared to be present as the rutile phase only, whilst the product P1 sample presented only anatase phase. All samples showed some peak broadening, indicative of the presence of nano-sized crystallites, as previously observed by electron microscopy. Previous research has indicated that nTiO_2_ phase has impacted upon toxicity towards biota,^22,63^ hence, such information is important to fully evaluate outcomes of toxicity testing.

In accordance with previous studies, variation in physical properties of extracted particles is observed.^9,38^ For example, primary nTiO_2_ particles extracted from consumer products S1 and S2 showed significant differences in morphology (Fig. 1B and C), despite both being utilised for UV protection in sunscreen formulations. These results emphasize the difficulty researchers face in the field of nano-ecotoxicology in selecting appropriate NMs for investigation, where NMs belonging to the same class of material vary extensively in physicochemical properties which will inevitably alter their fate and behaviour in the environment. As a result, it is difficult, or impossible, to effectively compare between studies utilising such materials. This issue is exacerbated by the fact that for >50% of NMs used commercially, the chemical structure is unknown.^4^ Additionally, other components of the product formulation may be difficult to separate from nanoparticles during the extraction process.^64^ In studies carried out by Philippe *et al.* (2018), all nTiO_2_ extracted from sunscreen was believed to be coated, most commonly with Al or Si.^9^ However, herein, based on EDS mapping, primary particles appeared to be present as pure TiO_2_. Product P1 additionally showed the presence of secondary particles – large micron-sized spheres (Fig. 1D) composed of SiO_2_ (from EDS measurements, Fig. S1Dii†), a common filler used in cosmetics. On the other hand, S1 showed the additional presence of hollow carbon-based organic spheres (Fig. 1B; EDS, Fig. S1B†), believed to be micellar structures from additional organic components present in the sunscreen which were not completely removed during extraction. No alumina (Al_2_O_3_), another common filler in cosmetics, was found during EDS measurements of all samples. Efforts must be directed at producing materials for ecotoxicological research that are representative of those that are likely to enter the natural environment, such as those displayed here from common user products.

### 3.2 Investigating the toxicity of research-grade and consumer nTiO_2_ on the marine cyanobacterium *Prochlorococcus*

**Fig. 2:**
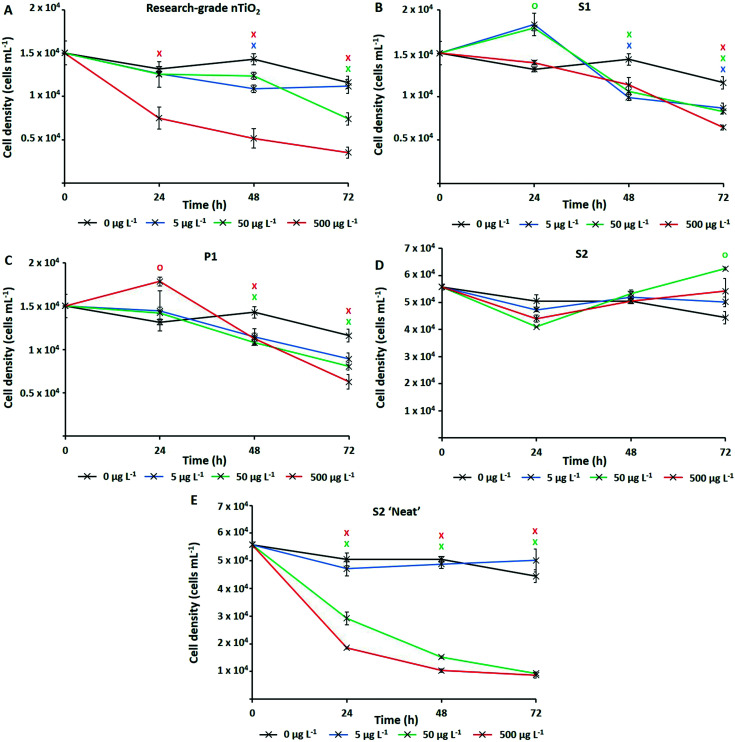
Cell density of *Prochlorococcus* MED4 when exposed to nTiO_2_ (0–500 μg L^−1^) for a period of 72 h under simulated natural conditions as measured by flow cytometry; A – research-grade nTiO_2_ nanopowder purchased from Sigma Aldrich; B – nTiO_2_ extracted from Skinceuticals™ sunscreen (S1); C – nTiO_2_ extracted from The Body Shop™ liquid foundation (P1); D – nTiO_2_ extracted from Boots Soltan™ sunscreen (S2); E – ‘Neat’ Boots Soltan™ sunscreen immersed in NSW. Data points are presented as the mean ± standard error (*n* = 3). Markers indicate where two-way *t*-tests revealed cell density of treated groups to be significantly lower (crosses) or higher (circles) than the untreated control at each timepoint (*p* ≤ 0.05).

The numerically most abundant phototroph on Earth, the marine cyanobacterium *Prochlorococcus*, was grown in the presence of research-grade nTiO_2_ (Sigma Aldrich) and nanoparticles extracted from common consumer products, as well as ‘neat’ sunscreen under environmentally relevant conditions, *i.e.*, natural oligotrophic seawater with relevant cell density (∼10^4^ cells mL^−1^) and nTiO_2_ concentrations (1, 50 and 500 μg L^−1^). Following 72 h exposure under simulated natural conditions (Fig. 2), significant declines in population size were experienced by *Prochlorococcus* strain MED4 in response to three out of four nTiO_2_ treatments when compared to the untreated control (*i.e.*, research-grade nTiO_2_, and nTiO_2_ extracted from S1 and P1, two-way *t*-test; *p* ≤ 0.05). Despite previous research indicating a key role of TiO_2_ phase in determining toxicity,^22,63^ no clear influence of varying nTiO_2_ phase was observed. Toxic effects were recorded in both rutile and anatase treatments, as well as mixed phase nTiO_2_. It must be acknowledged that due to the methods used to extract nanoparticles, materials may not accurately reflect those released into the environment within product matrixes. Here, behaviour of nanoparticles may be altered by the presence of other components of the product formulation, which require consideration. The process of nanoparticle release from consumer products upon their entry into the environment requires further investigation to comprehensively evaluate their impact.

Upon comparing the response displayed by cultures exposed to research-grade and extracted materials, recorded cell decline of *Prochlorococcus* was greatest during exposure to research-grade nTiO_2_ at higher concentrations (*i.e.*, 50 and 500 μg L^−1^). Here, exposure resulted in a significant reduction in cell density of up to 70% compared to the control after 72 h (two-way *t*-test, *p* ≤ 0.05), recorded in the 500 μg L^−1^ treatment (Fig. 2A). Significant declines were also recorded in the extracted S1 and P1 treatments; however, these were less severe, resulting in decreases in cell density of up to 46%, and 56% compared to the untreated control respectively (two-way *t*-test, *p* ≤ 0.05), at the highest concentration (500 μg L^−1^) (Fig. 2B and C). This result differs from trends described in previous works in literature, where materials extracted from sunscreen and toothpaste were recorded to exert stronger adverse effects than pristine research-grade nTiO_2_.^6^ In that work, it is likely that residual components of product formulations, not fully removed during the extraction process, are toxic at the relatively high concentrations which were tested, are toxic at the relatively high concentrations which were tested (1–5 mg L^−1^).^6^ Whilst, the highest concentration (500 μg L^−1^) tested here is greater than that predicted in the environment,^15^ some evidence of *Prochlorococcus* decline was also observed at 50 μg L^−1^. This concentration does not far exceed the concentration of TiO_2_ derived from sunscreen recorded in areas of high tourism within the natural environment (7–40 μg L^−1^).^17^ Therefore, potential exists for such materials to exert an adverse effect upon marine cyanobacteria such as *Prochlorococcus* in those areas more susceptible to localised pollution.

No adverse effect of exposure was recorded in response to S2 extracted nanoparticles. In fact, after 72 h average cell density of cultures exposed to S2 nanoparticles reached slightly higher values than control cultures (Fig. 2D). Interestingly, herein, cultures exposed nTiO_2_ extracted from S1 and P1 also displayed evidence of enhanced growth during early stages (24 h) of experimentation (Fig. 2B and C). Here, an approximate 10% increase in cell density of both the S1 and P1 treatment was observed relative to the untreated control following 24 h exposure (two-way *t*-test, *p* ≤ 0.05). A beneficial effect of some sunscreen formulations upon phytoplankton growth has been observed in previously published research.^8^ Such increases in cell density may arise from possible biostimulating effects of residual product components such as antioxidants, preservatives and moisturisers, believed able to enhance or stimulate growth of phytoplankton;^8^ or essential nutrients such as N, P and Si, reportedly released by sunscreen upon entry into seawater.^17^

Whilst evidence of significant cell decline was observed in the presence of both research-grade and consumer nTiO_2_ (after 72 h), these declines were not as severe as those experienced by cultures exposed to ‘neat’ sunscreen S2 immersed in seawater (Fig. 2E). Here, decline of the cyanobacterial population was the most rapid, and resulted in an 81% decrease of the population by the end of the 72 h incubation at the highest concentration (500 μg L^−1^) relative to the untreated control (two-way *t*-test, *p* ≤ 0.05). However, given ‘neat’ sunscreen treatments where established based on estimated nTiO_2_ content, exact nTiO_2_ concentrations may be higher or lower than intended. Nevertheless, sunscreens containing nTiO_2_ have previously been recorded to exert toxicity upon phytoplankton *via* generation of ROS,^27^ which are particularly toxic to *Prochlorococcus* due to its lack of catalase.^65^ Given that no adverse effect was observed when exposing *Prochlorococcus* to nTiO_2_ extracted from sunscreen S2, it is likely that toxic effects arise from the other components of the sunscreen formulation which *Prochlorococcus* is highly sensitive to, such as organic compounds or metals.^66,67^ For example, the organic UV-filter octocrylene, found in many sunscreen products, is toxic to a range of marine species.^68^ These findings support the belief that whilst examining the effects of NMs utilised in consumer goods, understanding the exact chemical characteristics of materials is vital. It is important to consider any unknown components of product formulations which may too exert adverse (or stimulating) effects upon biota,^69^ or alter outcomes of toxicity testing. Without such information, we are unable to attribute potential toxicity solely to NMs.

### 3.3 Medium-term exposure of *Prochlorococcus* to research-grade nTiO_2_

**Fig. 3:**
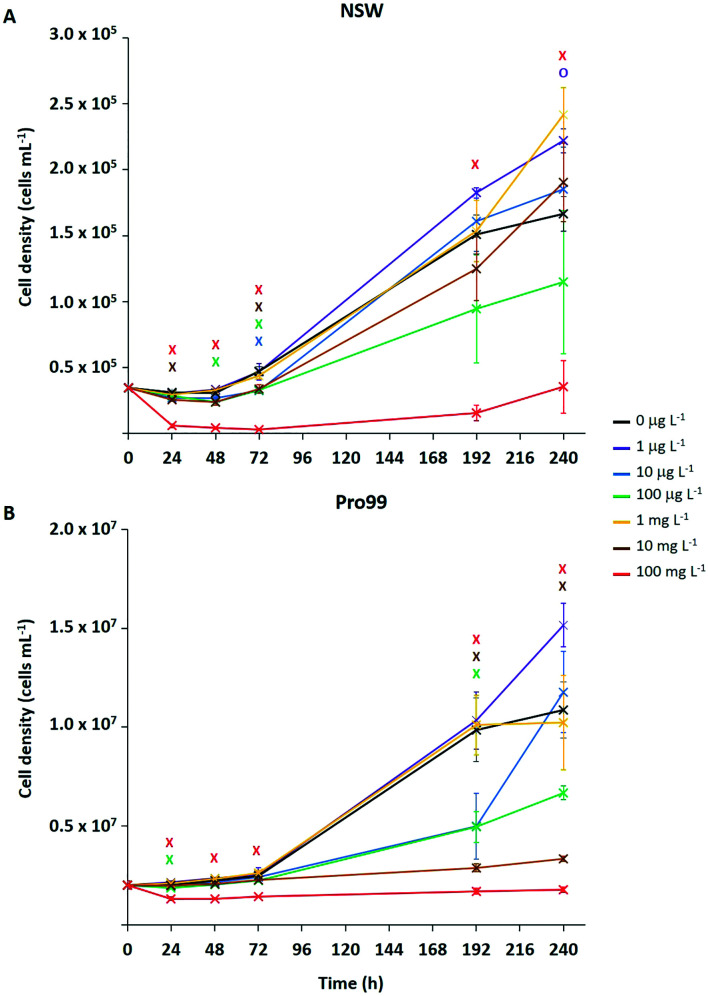
Medium-term exposure of *Prochlorococcus* MED4 to research-grade nTiO_2_ (Sigma Aldrich, 19.9 ± 6.6 nm (TEM)) in both NSW (A) and nutrient rich Pro99 media (B) at concentrations representing the environmental (0, 1, 10, 100 μg L^−1^) to supra-environmental range (1, 10, 100 mg L^−1^). Data points are presented as the mean ± standard error (*n* = 3). Markers indicate where two-way *t*-tests revealed cell density of treated groups to be significantly lower (crosses) or higher (circles) than the untreated control at each timepoint (*p* ≤ 0.05).

Typically, ecotoxicological studies are carried out for a number of days (3–4 d),^6,21–23,26–28,31,37^ thus missing the opportunity to assess chronic effects (>5 d) of a particular substance.^70^ However, examples of longer-term studies do exist.^71–73^ To address this and assess the ability of *Prochlorococcus* MED4 populations to recover from short-term (72 h) stress, incubations with research-grade nTiO_2_ observed to exert strongest effects during short-term exposure, were extended to 10 d (Fig. 3). These nanoparticles were identified as mixed rutile and anatase phase by P-XRD (see section 3.1) and hence represent all TiO_2_ phase types investigated. In this work, the nTiO_2_ concentrations examined were altered to span the predicted environmental (μg L^−1^) and supra-environmental range (mg L^−1^); hence, results are not directly comparable to short-term (72 h) experiments. Additionally, we tested cultures grown in nutrient rich Pro99 media to assess the effect of altered experimental conditions. When monitoring cultures exposed to research-grade nTiO_2_ in the mg L^−1^ range, deposited material was visible in the culture flasks, which we suggest to be hetero-aggregations of nTiO_2_ and cyanobacterial cells (NP–cell). Due to precipitation arising from increased density, any cells entrapped in NP–cell aggregates would likely be removed from the water column, thus reducing the planktonic cyanobacterial population. Given this, only freely suspended *Prochlorococcus* cells were used for the calculation of cell density, although NP–cell aggregates were also monitored.

Significant declines in the free-living *Prochlorococcus* population were observed at a range of concentrations of added nTiO_2_ ≥10 μg L^−1^ at specific timepoints throughout the 10 d incubation in both NSW and Pro99 media (see Fig. 3). In NSW (Fig. 3A), at the 24 h timepoint only the 10 and 100 mg L^−1^ treatments caused a significant decline in cell density compared to the untreated control (two-way *t*-test, *p* ≤ 0.05). After 48 h the 100 mg L^−1^ continued to exert significant declines in the cyanobacterial population, additionally the cell density of the 10 μg L^−1^ treatment was also significantly lower than the untreated control (two-way *t*-test, *p* ≤ 0.05). Following 72 h exposure, significant declines were recorded in the 10 μg L^−1^, 100 μg L^−1^, 10 mg L^−1^ and 100 mg L^−1^ treatments (two-way *t*-test, *p* ≤ 0.05). However, in later stages of exposure (*i.e.*, 192 and 240 h) only 100 mg L^−1^ nTiO_2_ caused a significant decline in the *Prochlorococcus* population in NSW compared to control cultures (two-way *t*-test, *p* ≤ 0.05). Cultures grown in nutrient rich Pro99 media (Fig. 3B) experienced a decrease in cell density compared to the control in response to 100 mg L^−1^ nTiO_2_ at the each timepoint 24–72 h (two-way *t*-test, *p* ≤ 0.05). Additionally, at the 24 h timepoint, the 10 μg L^−1^ treatment also caused a significant decline in the cyanobacterial population (two-way *t*-test, *p* ≤ 0.05). In later stages of exposure (*i.e.*, 192–240 h) cultures grown in Pro99 media experienced significant declines in cell density in response to 10 and 100 mg L^−1^ nTiO_2_ compared to the untreated control, where at the 192 h timepoint 10 μg L^−1^ was also observed to significantly reduce cell number (two-way *t*-test, *p* ≤ 0.05).

Adverse effects in terms of cell decline were augmented when concentrations were increased to those in the supra-environmental range (*i.e.*, 100 mg L^−1^, see Fig. 3A and B), likely due to increased rate of encounter between nanoparticles and cyanobacteria. Following 10 d, exposure to 100 mg L^−1^ nTiO_2_ drove cell declines of 79% and 84% in NSW and Pro99 media when compared to the untreated control respectively (two-way *t*-test, *p* ≤ 0.05). Cultures grown to higher cell densities in nutrient-rich media were observed to suffer greater adverse effects of exposure than those grown in NSW (see Fig. 3B). This likely arises due to an increased rate of encounter between nTiO_2_ and cyanobacteria in cell-dense cultures. Unexpectedly, no negative effect was observed at the concentration of 1 mg L^−1^ in NSW or Pro99 media, possibly representing an anomalous result. This being said, evidence of increased growth following incubations with metal oxide NMs has previously been recorded in phytoplankton exposed to nCeO_2_ (<5 mg L^−1^).^26,74^

Despite evidence of early declines (<72 h) in cell number displayed by *Prochlorococcus* following exposure to research-grade nTiO_2_ (*i.e.*, ≥10 μg L^−1^ and up to 72 h), the ability of the cyanobacterial culture to overcome initial stress in NSW was revealed in extended incubations when exposed to both environmentally relevant and supra-environmental concentrations ≤10 mg L^−1^ (Fig. 3A). The recovery of microbial populations following metal oxide NM exposure has recently been reported.^70,72,73^ For example, decline of *Picochlorum* sp. during early exposure in response to nTiO_2_ and ZnO nanoparticles (10 mg L^−1^) was reversed in later stages, believed due to aggregation and sedimentation of particles, thus reducing direct exposure of phytoplankton to stable non-sedimenting particles.^73^ Herein, growth of *Prochlorococcus* was positive in the majority of treatments investigated, despite being significantly lower than the untreated control at specific concentrations, particularly in early stages. This suggests that although metal oxide NMs such as nTiO_2_ may remove a fraction of the microbial population through processes of aggregation and sedimentation, the remaining planktonic population continues to grow and is able to recover to ambient cell densities in extended exposure.

### 3.4 Physical toxicity and entrapment of cyanobacteria by nTiO_2_

**Fig. 4:**
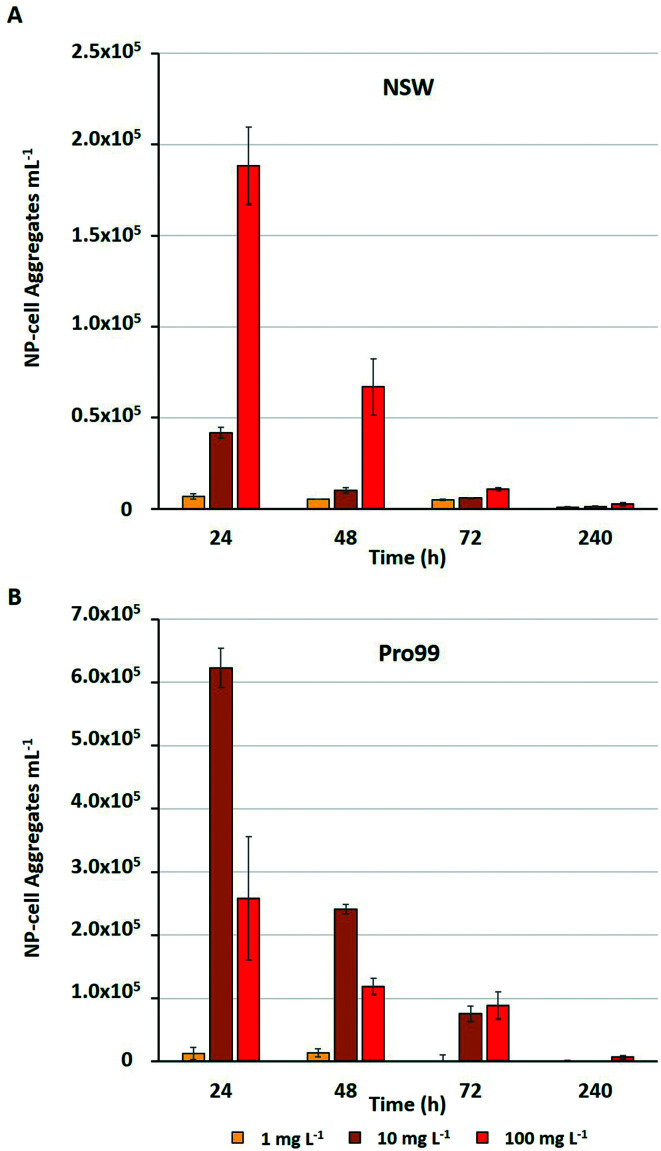
Estimated number of NP–cell aggregates mL^−1^ comprised of nTiO_2_ and *Prochlorococcus* MED4 observed during flow cytometric analysis of *Prochlorococcus* MED4 exposed to nTiO_2_ (Sigma Aldrich) in A) NSW, and B) Pro99 media for a period of 240 h (*n* = 3).

Previous research has highlighted that aggregation between metal oxide NMs and phytoplankton is widespread during laboratory exposure.^23–25,32,75^ The physical interaction between NMs and cells, and their entrapment by aggregates is often considered the primary driver of adverse effects experienced by marine microbial organisms.^23,25,36,75^ Adherence of metal oxide NMs to the cell surface is believed to enhance oxidative stress by increasing ROS production^33,76^ and may cause physical damage to the cell wall or membrane.^75^ Aggregation is also proposed to reduce light availability in water, although such shading effects have not been conclusively proven.^34,37,77^ Through the subsequent process of sedimentation, hetero-aggregation of NMs and cells effectively removes phytoplankton from the water column, and this is believed to be the primary cause of cell decline in the work presented herein. During flow cytometric analysis of cyanobacterial cultures, it was possible to estimate the number of NP–cell aggregates present within samples in mg L^−1^ treatments (Fig. 4).

The estimated number of NP–cell aggregates was recorded to decline rapidly within the early stages of incubation, indicating their rapid settling and hence removal of cyanobacteria from the water column. In NSW (Fig. 4A) the number of aggregates in the 100 mg L^−1^ treatment declined by 64% between 24 and 48 h. This rapid precipitation and hence decrease in estimated NP–cell aggregate number was also observed in Pro99 supplemented cultures (Fig. 4B), and as expected the total aggregate count far exceeded those recorded in NSW, likely due to increased rate of encounter between nTiO_2_ and cyanobacteria at higher cell densities. In previous work, this trend has also been observed, with highest rates of aggregation associated with highest cell density and maximised detrimental effect to cells.^34^ Such increased rates of aggregation and interaction between nTiO_2_ and cyanobacteria may have driven the enhanced decline in cell number observed in cell-dense cultures observed in this study. However, findings vary, and it has also previously been recorded that aggregation and subsequent sedimentation of metal oxide NMs can reduce the negative effects associated with exposure.^37^ This process may explain the patterns of *Prochlorococcus* recovery observed when exposures were extended in NSW, where following aggregation and subsequent sedimentation of the majority of nTiO_2_, free-living cyanobacterial populations are able to recover.

**Fig. 5:**
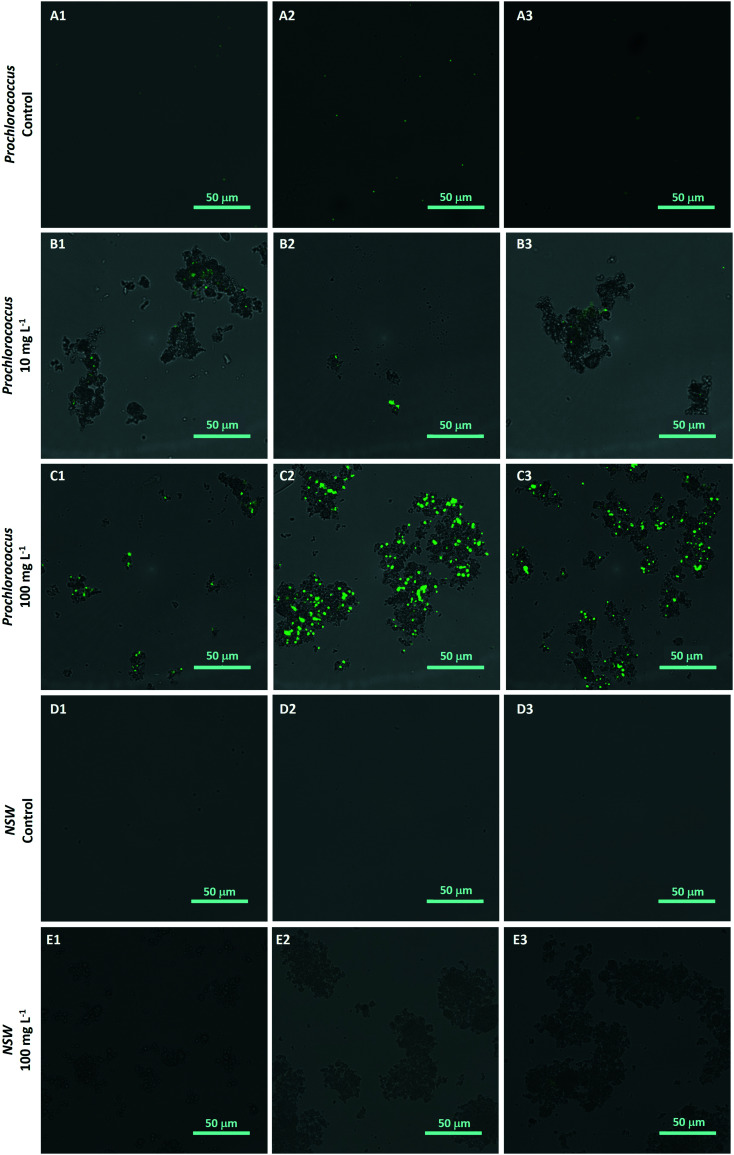
Hetero-aggregation of *Prochlorococcus* MED4 (stained with SYBR Gold (green)) and research-grade nTiO_2_ (Sigma Aldrich; non-stained particles) as observed by fluorescent microscopy; A1–3: untreated *Prochlorococcus* culture; B1–3 *Prochlorococcus* culture exposed to 10 mg L^−1^ nTiO_2_; C1–3 *Prochlorococcus* culture exposed to 100 mg L^−1^ nTiO_2_; D1–3 natural seawater control (no nTiO_2_); E1–3 natural seawater nTiO_2_ suspension (100 mg L^−1^). Images represent those merged from GFP and Brightfield channels.

The hetero-aggregation and subsequent entrapment of *Prochlorococcus* by nTiO_2_ was confirmed using fluorescent microscopy (Fig. 5). Here, aggregates of nTiO_2_ and cells readily formed in both NSW and nutrient-rich media, observed by microscopy at concentrations of 10 and 100 mg L^−1^. Control images obtained from NSW and *Prochlorococcus* samples in the absence of nanoparticles, supported the belief that precipitated material observed was indeed nTiO_2_, rather than other particulate matter. The entrapment of phytoplankton by nTiO_2_ has previously observed using microscopic techniques in work carried out with *P. tricornutum* in response to concentrations similar to those used in our study (50 mg L^−1^).^25^ Hetero-aggregates were recorded to reach sizes in the micron-range when measured along their longest axis (2.4–133.5 μm, ImageJ analysis), and displayed an average size of 28.0 μm (*n* = 100). Such aggregations of research-grade nTiO_2_ (25 nm) have been previously recorded to form within 30 minutes of addition into saline f/2 media within this concentration range.^32^

In response to NM exposure, marine phytoplankton have been observed to enhance the production of exopolymeric substances (EPS).^78–82^ The secretion of EPS is associated with microbial stress and believed a defence mechanism against NMs.^78^ For example, EPS was shown to protect the freshwater cyanobacterium *Synechocystis* PCC6803 from nTiO_2_-mediated stress by quenching toxic ROS species and reducing direct contact of nanoparticles with the cell surface.^81^ Whilst cyanobacteria are able to produce EPS, *Prochlorococcus* lacks most EPS related proteins, believed lost through the evolutionary streamlining of the *Prochlorococcus* genome due to energy costs of EPS production likely outweighing any benefits.^83^ As such, any enhancement to EPS production displayed by *Prochlorococcus* during exposures is likely to be limited, and is not believed to be an influential factor in the hetero-aggregation process proposed. However, as the presence of EPS is believed likely to enhance aggregation of nTiO_2_ and hence reduce its bioavailability over time,^82^ any increase in EPS production may act to promote the processes of entrapment and co-precipitation described. Indeed, while EPS was recorded to mitigate toxicity in *Synechocystis*, hetero-aggregation between the EPS-producing cyanobacteria and nTiO_2_ was still observed.^81^

In the natural environment, processes of entrapment and sedimentation may reduce the availability of phytoplankton prey,^35^ potentially causing an energy deficit in higher trophic levels. Additionally, through ingestion of entrapped phytoplankton, or indirect ingestion during feeding, trophic transport and possible bioaccumulation of metal oxide NMs such as nTiO_2_ may occur. Evidence of trophic transfer of nTiO_2_ from marine invertebrates to fish has been observed under laboratory conditions.^84^ Once ingested, metal oxide NMs may exert adverse effects upon higher organisms, such as disruption to immune processes and antioxidant activities, as has been recorded in marine invertebrates following exposure to nCeO_2_.^85,86^ Given that nTiO_2_ has been recorded to interact with other contaminants within the water column including tributyltin, phenanthrene, polycyclic aromatic hydrocarbons; enhancing their uptake and toxicity to biota,^87,88^ it is important to also consider the potential of metal oxide NM aggregates to adsorb such pollutants which may also be passed to higher trophic levels of the marine food web.

### 3.5 Behaviour of nTiO_2_ in seawater

The fate and behaviour of NMs upon entry into the environment largely determines their bioavailability and mechanism of toxicity towards biota, and is therefore a key consideration during nano-ecotoxicological research.^73,75,82^ Upon entry into saline media with high ionic strength (∼0.7 eq L^−1^),^89^ metal oxide NMs have generally been observed to aggregate freely and undergo sedimentation, at a faster rate than bulk material.^36,89^ Herein, we utilised dynamic light scattering (DLS) methods to confirm that research-grade nTiO_2_ (1 and 100 mg L^−1^) displays rapid aggregation upon entry into saline media. Hence, supporting our belief that the processes of nTiO_2_ aggregation and subsequent co-precipitation with cells is the key driver of cyanobacterial decline recorded in toxicity experiments described above (sections 3.3 and 3.4).

DLS revealed that nTiO_2_ reached sizes far exceeding those of primary particles analysed by TEM (19.9 ± 6.6 nm), displaying an average size of 1547–6560 nm throughout the 14 d experiment (Table 2). These sizes are expected to be somewhat larger than those recorded by TEM (19.9 ± 6.6 nm), since dynamic light scattering measures the hydrodynamic size of colloidal nanoparticles, taking into account van der Waals and interparticle interactions. However, the sizes observed for particles in NSW are far larger than would be expected due to their colloidal behaviour, indicating their rapid aggregation. It should be noted that supra-environmental concentrations have been investigated here due to the poor sensitivity of the equipment to the lower, environmentally relevant concentrations. Nanoparticles were recorded to aggregate immediately upon entry into NSW and DLS measurements displayed that variation in *z*-average size between replicates was large, suggesting a high variability in aggregate size and low uniformity of this process. This is reflected in the polydispersity index (PDI) values, which remained in the range of 0.58–1.00 throughout the 14 d experiment observed, indicating the presence of a largely polydisperse system. It must be noted, though, that due to biases towards larger particles through use of DLS analysis, smaller particles are likely under-represented.^90^ Precipitation of nTiO_2_ from the water column as a result of increased aggregation was recorded, and deposits of nTiO_2_ were visible at the bottom of flasks after 24 h incubation with media and throughout the remaining timepoints of the experiment (see Fig. S4†).

**Table 2:** Summary of results obtained from DLS analysis of research-grade nTiO_2_ (Sigma Aldrich, 19.9 ± 6.6 nm (TEM)) suspensions in NSW

Time (h)	1 mg L^−1^		100 mg L^−1^	
	*Z*-Average size (d.nm)	Polydispersity index (PDI)	*Z*-Average size (d.nm)	Polydispersity index (PDI)
0	1580 ± 124	0.804 ± 0.170	1624 ± 113	0.580 ± 0.156
1	5626 ± 586	1.000	6560 ± 464	1.000
2	2262 ± 129	0.890 ± 0.191	4994 ± 198	1.000
4	1393 ± 284	0.907 ± 0.081	1569 ± 129	0.737 ± 0.108
24	2367 ± 665	1.000	5636 ± 670	0.980 ± 0.035
48	2253 ± 519	1.000	4133 ± 956	0.957 ± 0.055
72	2741 ± 670	1.000	1966 ± 936	0.978 ± 0.025
168	Undetectable		1994 ± 141	1.000
240	Undetectable		1564 ± 168	0.953 ± 0.060
336	Undetectable		3133 ± 215	1.000

The mean *z*-average size (d.nm) of nTiO_2_ at *T*_0_ was 1580 and 1624 nm for 1 and 100 mg L^−1^ samples respectively, indicating the rapid aggregation of nTiO_2_ upon entry into NSW. The PDI at the start of the experiment also indicated a high level of aggregation between nTiO_2_ particles, 0.804 and 0.580 for 1 and 100 mg L^−1^ samples, respectively. Over the first hour, aggregation of nTiO_2_ was most rapid, reaching respective peaks in *z*-average size of 5626 and 6560 nm in the 1 and 100 mg L^−1^ treatments, whilst PDI reached a value of 1.000. Following this, *z*-average size in the 1 mg L^−1^ group appeared to show a slight decrease for subsequent timepoints, although variation remained high, likely indicating that particles are aggregating and sinking, with larger particles no longer within the measurement window of the DLS. PDI values remained high during this period, with a value of 1.000 observed at 24, 48 and 72 h. After 72 h, samples at the 1 mg L^−1^ concentration were undetectable and therefore unsuitable for data acquisition by DLS. The removal of nTiO_2_ from the water column *via* precipitation, observable in flasks after 24 h (see Fig. S3†), and hence reduction in suspended nTiO_2_ particles is believed to cause the lack of data acquisition at these timepoints following 72 h during DLS measurement at the 1 mg L^−1^ concentration.

Mean *z*-average size of nTiO_2_ added to NSW at the 100 mg L^−1^ concentration varied throughout the 14 d experiment but displayed clear evidence of extensive aggregation of nTiO_2_, also indicated by the high PDI values recorded. Overall, *z*-average size varied 1547–6560 nm during the experiment. Hence, the aggregation of nTiO_2_ within NSW appears a rapid, non-uniform and highly variable process. As with lower concentration samples, a large extent of deposition of nTiO_2_ aggregates were visible after 24 h and continued throughout the 14 d experiment (see Fig. S3†). In later stages (>72 h), mean *z*-average size appeared to show a slight decrease. Such data suggests that the size of aggregates remaining in suspension during later stages of the experiment were smaller in size as larger aggregates had likely undergone precipitation and were deposited at the bottom of flasks. Despite the clear evidence of extensive aggregation and sedimentation behaviour of nanoparticles recorded, it should be noted that these observations were made at concentrations considerably higher than those predicted in the environment (due to the low sensitivity of equipment to μg L^−1^ concentrations). As such, at lower environmental concentrations the rate of homo-aggregation is likely to be reduced, due to effects of dilution and a decreased rate of encounter between individual particles.

Additional information regarding nTiO_2_ sedimentation was obtained during flow cytometric analysis by recording the event number of nTiO_2_ of varying concentration in NSW in the absence of cyanobacteria (see Section S2.1 and Fig. S3†). Here, particularly in the mg L^−1^ range, nTiO_2_ was observed to undergo rapid sedimentation following entry into seawater, correlating with previous research^32,36,37,89^ and the results acquired by DLS. Up to 58% loss of nTiO_2_*via* precipitation within 6 h has previously been observed using UV-vis spectrophotometry.^32^ Herein, nTiO_2_ counts were recorded to decrease 57%, 81% and 89% in 1, 10 and 100 mg L^−1^ samples respectively during the initial 24 h of entry into NSW. Following 48–72 h, the number of nTiO_2_ aggregates observed in suspension during our work was comparable between all concentrations.

It is clear from DLS and flow cytometry experiments that rapid aggregation and sedimentation of nTiO_2_ occurs in marine water. As a result, the bioavailability of nTiO_2_ towards planktonic organisms displays a continual decrease, as has been recorded previously.^82^ As such, the risk of nTiO_2_ towards marine microbial species appears lowered during extended exposure. However, at high concentrations, negative effects of exposure may be experienced for longer periods as populations take time to recover or nTiO_2_ takes longer durations to enter lower fractions of the water column. In the natural environment the process of reduced bioavailability will likely be concentration-specific, with a lowered rate of encounter between individual nTiO_2_ particles at lower concentrations and hence likely lowered homo-aggregation. Extent of sedimentation observed will also be influenced by other environmental variables not investigated in detail here, including pH, and the presence of natural organic and particulate matter.^91^ However, despite the high levels of sedimentation recorded in this study, previous environmental assessment by Gondikas *et al.* (2018) has revealed nTiO_2_ derived from seasonal use of sunscreen remain in suspension for a period of weeks before settling, where particles may interact with planktonic organisms.^13^

### 3.6 Identifying molecular features of nTiO_2_ toxicity: shotgun proteomic analysis

Alongside observations of cell decline, and direct physical entrapment of cyanobacteria, we performed a shotgun proteomic analysis of *Prochlorococcus* to inform on any other potential toxic effects on this phototroph other than physical entrapment in NM aggregates. Here, exposure to research-grade nTiO_2_ did not produce metabolic alterations in *Prochlorococcus*, such as oxidative and nutrient stress or reduction in photosynthetic machinery.

A number of toxic modes of action have been proposed for metal oxide NMs against microbial species, although largely these have been performed at much higher concentrations. Oxidative stress due to the photocatalytic generation of reactive oxygen species (ROS) by nTiO_2_ is believed a key driver of stress.^21,32,92,93^ Presence of UV light is therefore a key consideration in experimental design,^27^ hence in our study full spectrum bulbs with environmentally-relevant levels of UV were utilised. Environmental levels of UV have been previously recorded to induce phototoxic effects of nTiO_2_ against phytoplankton with ROS concentration increasing with increased nTiO_2_ concentration.^21^ Therefore, it can be proposed that oxidative stress due to ROS production may be a feature of toxicity observed herein during incubations with nTiO_2_. Photosynthetic processes have also been observed to be disrupted in response to nTiO_2_, such as decreased oxygen generation^37^ and damage to photosystems.^94^ Metal oxide NMs such as ZnO have been recorded to cause toxicity due to the release of toxic Zn ions,^31,95,96^ however the release of dissolved Ti from nTiO_2_ is believed to be negligible under experimental conditions.^23,97,98^ Additionally, TiO_2_ has been reported to absorb Zn and P from experimental media,^99^ as well as to effectively reduce total N and P during microcosm experiments carried out in freshwater.^34^

Cultures were exposed to an environmentally relevant concentration (100 μg L^−1^) of research-grade nTiO_2_ previously recorded to induce a decline in average cell density in both cell dense and ambient cell densities, for a period of 24 h (7–8% decline (24 h), see Fig. 3). Here, experiments were carried out using cell-dense cultures only grown in Pro99 media to ensure sufficient biological material was collected. Following analysis of both cellular and extracellular proteomes, little difference was observed between treated and control samples (Fig. S5†). Indeed, no individual proteins were significantly altered in abundance following the addition of nTiO_2_. Protein function was classified using the Uniprot database and KEGG assignment. Here, little difference was also apparent upon classifying proteins based upon their biological function (see ESI† data Tables S1 and S2, and Fig. S6). The cellular proteome was largely comprised of proteins associated with basic cellular processes, making up ∼33% of proteins identified. Proteins involved with central metabolic processes were also relatively abundant in cellular proteome samples and made up ∼20% of proteins identified in control and nTiO_2_-treated cultures. Despite previous research suggesting disruption to photosynthesis and induction of oxidative stress pathways is likely associated with nTiO_2_ exposure, evidence of such effects was not recorded. Herein, proteins associated with photosynthetic processes and oxidative stress represented approximately 6% and 3% of the cellular proteome respectively. Similarly, any evidence of nutrient stress was not apparent. In the extracellular proteome, nTiO_2_ exposure was associated with a slight decrease in membrane transport proteins (40% (control) to 36% (nTiO_2_)), and slight increase in proteins associated with basic cellular processes (20% (control) to 22% (nTiO_2_)) and metabolism (17% (control) to 21% (nTiO_2_)). The increase in cytoplasmic-associated proteins may be indicative of a slight increase in cell lysis in treated samples, which may result from hetero-aggregation and entrapment by nTiO_2_. Based on the proteomic data reported here, it can be suggested that molecular features of nTiO_2_ toxicity may be negligible at the population scale when exposed to environmentally relevant concentrations. Rather, it appears cell decline is primarily driven by physical effects of exposure.

### 3.7 Effects of consumer nTiO_2_ upon natural marine communities

Given the complex nature of the marine microbial community, it is important to consider the community-wide response towards environmental contaminants. Exposure of a natural marine microbial community to nTiO_2_ extracted from sunscreen (S2) and ‘neat’ nTiO_2_-containing sunscreen (S2) immersed in seawater at an environmentally relevant concentration of 25 μg L^−1^,^17^ revealed negligible effects of nTiO_2_ exposure upon community composition under environmental conditions. Rather, any observed alterations in community structure were primarily associated with the presence of ‘neat’ sunscreen, and likely attributed to the other components of the product formulation.

**Fig. 6:**
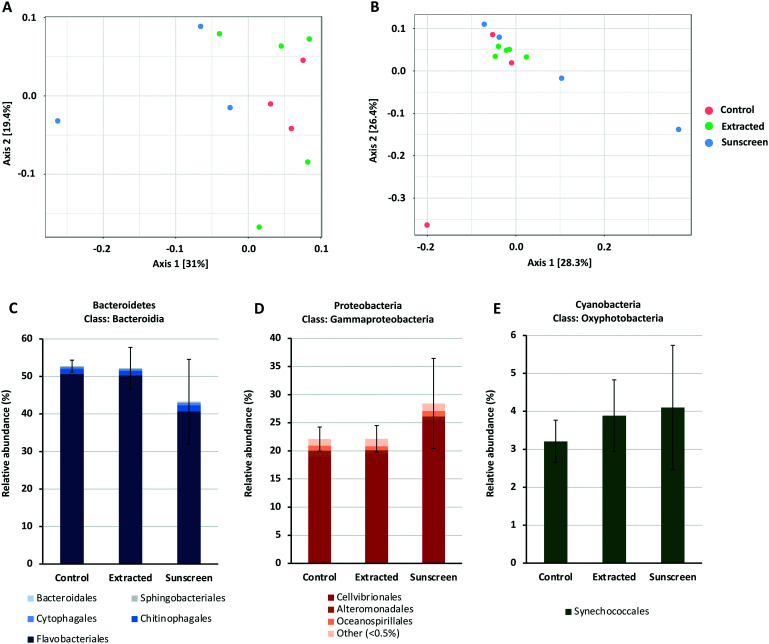
PCoA plot of 16S rRNA (A) and 18S rRNA (B) data based on Bray–Curtis dissimilarity. Coloured circles indicate samples belonging to each treatment; red circles represent control samples, where no nTiO_2_ was added; green circles represent samples treated with nTiO_2_ extracted from sunscreen (S2); blue circles represent samples treated with ‘neat’ sunscreen. PERMANOVA analysis was performed on both datasets respectively; 16S rRNA: *F*-value = 1.4814, *R*-squared = 0.27025, *p*-value < 0.06; 18S rRNA: *F*-value = 1.2109, *R*-squared = 0.21203, *p*-value < 0.144. Panel A and B were adapted from output generated by MicrobiomeAnalyst software. Bar charts presented in panels C–E display the average relative abundance (%) of the Bacteroidia, Gammaproteobacteria and Oxyphotobacteria classes in terms of individual taxonomic orders in each of the treatments respectively.

Amplicon sequencing data for bacterial and eukaryotic communities exposed to either treatment was compared to that obtained from of an untreated control where no nTiO_2_ or sunscreen was added. Principle coordinates analysis based on Bray–Curtis dissimilarity, in conjunction with PERMANOVA analysis (Fig. 6A and B), revealed no significant differences in the 16S rRNA bacterial or 18S rRNA eukaryotic community composition at the ASV level between individual treatments. Similarly, measures of species richness and evenness for both 16S and 18S rRNA datasets, displayed no statistical variation between treatments (see Tables S2 and S3†).

To gain additional insight into the composition of bacterial and eukaryotic communities, the relative abundance of major bacterial and eukaryotic phyla present in control and treated samples (25 μg L^−1^) was calculated (see Fig. S7 and S8†). Here, bacterial communities were dominated by the Bacteroidetes and Proteobacteria phyla, making up approximately 90% of the total community (Fig. S7†). In the ‘neat’ sunscreen treatment the proportion of Bacteroidetes to Proteobacteria appeared to differ from both the untreated control and nTiO_2_ treatments. In control and extracted nTiO_2_-exposed samples average relative abundance of Proteobacteria and Bacteroidetes was approximately 41% and 52–53% respectively, compared to 48% and 44% in ‘neat’ sunscreen samples (Fig. 6C and D). However, due to large variation between individual samples belonging to the ‘neat’ sunscreen treatment this difference was not significant. Here, the increase in relative abundance of Proteobacteria in the sunscreen treatment was largely due to an increase in relative abundance of Gammaproteobacteria from 25–34%. Specifically, an increase in the Cellvibrionales order made up much of this change (Fig. 6D). The relative decrease in the Bacteroidetes phylum was largely attributed to a decline in relative abundance of the Flavobacteria in response to neat sunscreen, reducing on average from 51% in the untreated control to 41% (Fig. 6C). The reduction in the ratio of Bacteroidetes to Proteobacteria has previously been recorded in marine biofilm communities exposed to nTiO_2_-treated surfaces.^100^ Similarly, in riverine environments, Bacteroidetes members such as Flavobacteria have been recorded to display increased susceptibility to nTiO_2_ during community exposure.^101^ In this example, Actinobacteria also displayed a reduced relative abundance in the presence of nTiO_2_, whereas comparatively Betaproteobacteria were observed to increase by approximately 40% under the same conditions in comparison to the untreated control.^101^ These results were obtained at a test concentration of 100 mg L^−1^, far exceeding that measured in the environment.^17^ It is possible that should nTiO_2_ concentrations have been increased in this experiment, any decline in abundance of Flavobacteria in response to nTiO_2_-containing sunscreen would have been exacerbated and perhaps observed in the extracted nTiO_2_ treatment. However, in our study it appears these alterations arise as a result of other components of the sunscreen formulation rather than the action of nTiO_2_, as no such changes were observed in the extracted nTiO_2_ treatment. Despite *Prochlorococcus* displaying declines in cell density in earlier testing (sections 3.2 and 3.3), no significant effect on relative abundance of cyanobacteria was recorded in either treatment during community exposure. In fact, on average a slight increase in cyanobacteria was observed in both extracted nTiO_2_ and neat sunscreen treatments (Fig. 6E). Overall, only two bacterial phyla were observed to alter significantly in relative abundance between control and extracted nTiO_2_ or sunscreen-treated samples as a result of two-way *t*-tests. Both were observed in the extracted nTiO_2_ (S2) treatment and belonged to low abundant phyla; here, Fusobacteria displayed a significant increase in relative abundance from 0.007% in the control to 0.035% in the extracted S2 nTiO_2_ treatment (*p* = 0.043); whilst Lentisphaerae, reduced in relative abundance from 0.0015% in the control to 0.0011% in S2 nTiO_2_ samples (*p* = 0.024).

Eukaryotic communities were principally made up of phototrophic organisms belonging to the Ochrophyta, Prymnesiophyceae, Chlorophyta and Protalveolata phyla, with Arthropoda also relatively abundant in control and ‘neat’ sunscreen treatments (Fig. S8†). Species belonging to the Ochrophyta phylum were most abundant in all treatments, representing 28–30% of ASVs identified. The Ochrophyta largely comprises phototrophic organisms, and here diatoms made up the majority of taxa in this phylum and represented 24–25% of eukaryotic species identified across all treatments. Once again, differences in relative abundance of eukaryotic phyla between control and treated samples were tested by means of two-way *t*-tests. Here, no significant differences were identified despite evidence being available upon the negative effect of nTiO_2_ exposure towards eukaryotic phytoplankton in the literature, albeit largely recorded at greater concentrations (1–13 mg L^−1^).^6,21–30^ Previous research has revealed that at such concentrations, varying eukaryotic taxa display differential sensitivity to nTiO_2_ exposure.^21,27^ For example, Sendra *et al.* (2017) present that following exposure to nTiO_2_, and nTiO_2_-containing sunscreens the marine microalgae *Nannochloropsis gaditana* displays highest sensitivity to all treatments compared to the diatom, *Chaetoceros gracilis*, the dinoflagellate, *Amphidinium carterae*, and coccolithophore, *Pleuroochrysis roscoffensis*.^27^ Whilst such evidence exists, our results suggests that marine eukaryotic communities are little affected by exposure to environmentally-relevant concentrations of nTiO_2_ extracted from, or in the presence of nTiO_2_-containing sunscreen.

The findings presented suggest that current concentrations nTiO_2_ are unlikely to drive alterations to the structure and biodiversity of natural marine communities and is therefore unlikely to impact upon community functioning. Based on this, we can predict that the likely environmental risk of nTiO_2_ derived from consumer products such as sunscreen towards marine microbial communities is low. However, it remains that should contamination of the marine environment by NMs continue to increase, evidence suggests that phototrophic organisms may be negatively affected within hotspots of pollution comprising higher concentrations, mainly due to hetero-aggregation and removal from the water column *via* sinking.

## 4. Conclusions

Overall, we have shown that the interaction between marine microbial species and nTiO_2_ appears a highly dynamic process influenced largely by the behaviour of nanoparticles in saline media. Toxic endpoints appear dependent upon the length of exposure, where risks associated with nTiO_2_ exposure to marine microbial species appear low at currently predicted environmental concentrations. We have shown for the first time that the ecologically significant cyanobacterium, *Prochlorococcus*, suffers short-term (72 h) adverse effects following exposure to nTiO_2_, mainly due to hetero-aggregation with agglomerated nTiO_2_, and subsequent sinking out of the water column. Nevertheless, populations could recover when incubations were extended in natural oligotrophic seawater. No other sign of metabolic stress was observed by high throughput proteomics at a concentration relevant to those predicted in the environment, suggesting that physical interactions between nanoparticles and cyanobacteria are responsible for cell declines recorded. Monitoring of natural marine bacterial and eukaryotic communities exposed to nTiO_2_ and nTiO_2_-containing sunscreen, revealed no effect of either treatment at current environmental concentrations, suggesting that neither is likely to alter marine community structure or diversity in the natural environment.

The works presented here support the belief that the current environmental risk of nTiO_2_ is low, however uncertainties exist in the environmental concentrations of NMs currently predicted,^102^ and hotspots of contamination may serve to produce localised areas of cell decline given that severity of negative effects is enhanced with increasing nTiO_2_ concentration. The interaction and synergistic effect of NMs and other contaminants also requires attention given the high affinity of materials such as nTiO_2_ with other contaminants,^88,103,104^ which may act to enhance toxicity at lower concentrations. It is important that we continue to develop analytical techniques to reveal NM concentrations within the natural environment and enhance experimental work by studying materials that represent those likely to be entered into aquatic systems. As such, the use of extracted materials as we have examined in the above study provides great scope for future research in the field. However, there is a need to consider the whole product formulation in research on nano-enabled products, where specific components may play key roles in toxicity which are not identified when using uncoated nanoparticles. In particular, processes of NM release from product matrixes and their resultant state requires attention and is important to fully assess the environmental impact of engineered nanomaterials.

## Conflicts of interest

There are no conflicts to declare.

## Supplementary Material

EN-008-D0EN00883D-s001

EN-008-D0EN00883D-s002

EN-008-D0EN00883D-s003

EN-008-D0EN00883D-s004

EN-008-D0EN00883D-s005

EN-008-D0EN00883D-s006

EN-008-D0EN00883D-s007

EN-008-D0EN00883D-s008

EN-008-D0EN00883D-s009

EN-008-D0EN00883D-s010

EN-008-D0EN00883D-s011

EN-008-D0EN00883D-s012
