# Supplementary material for: Environmentally relevant concentrations of titanium dioxide nanoparticles pose negligible risk to marine microbes
Source: Environ Sci Nano. 2021 Apr 9;8(5):1236–55. doi: 10.1039/d0en00883d (PMC8136324; doi:10.1039/d0en00883d)
Supplement: EN-008-D0EN00883D-s002 [file EN-008-D0EN00883D-s002.pdf]

Electronic Supplementary Material (ESI) for Environmental Science: Nano.
This journal is © The Royal Society of Chemistry 2021

Environmentally relevant concentrations of titanium dioxide nanoparticles pose negligible risk to marine microbes

Craig J. Dedman,[a,b*] Aaron M. King,[c] Joseph A. Christie-Oleza,[a,d,e*] and Gemma-Louise Davies[c*]

[a] *School of Life Sciences, Gibbet Hill Campus, University of Warwick, Coventry, CV4 7AL, United Kingdom.*
[b] *Department of Chemistry, University of Warwick, Gibbet Hill, Coventry, CV4 7EQ, United Kingdom.*
[c] *UCL Department of Chemistry, University College London, 20 Gordon Street, London, WC1H 0AJ, United Kingdom.*
[d] *Department of Biology, University of the Balearic Islands, Ctra. Valldemossa, km 7.5. CP: 07122, Palma, Spain.*
[e] *IMEDEA (CSIC-UIB). CP: 07190, Esporles, Spain.*

[*] Corresponding authors: C.Dedman@warwick.ac.uk; joseph.christie@uib.eu; gemma-louise.davies@ucl.ac.uk.

Supplementary Information

SI.1 Characterisation of nTiO$_2$

SI.1.1 EDS analysis of material utilised in short-term toxicity tests

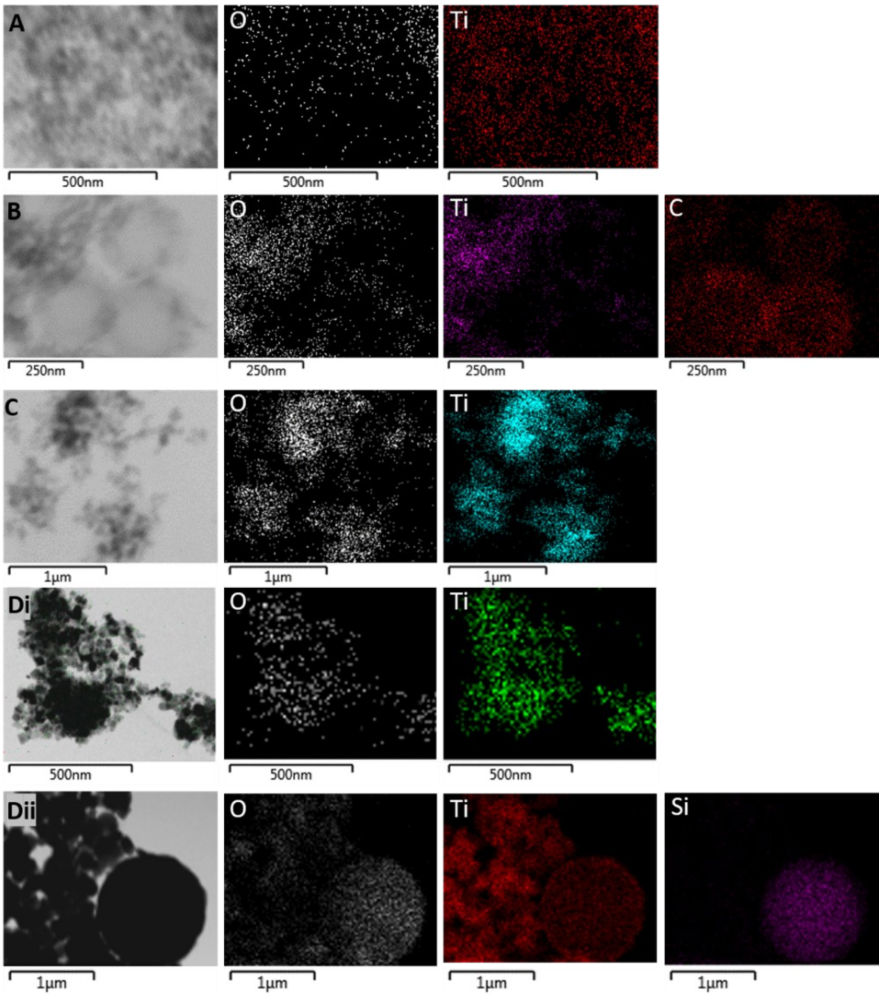

Figure SI.1. A – research-grade nTiO$_2$ nanopowder purchased from Sigma Aldrich; B – nTiO$_2$ extracted from Skinceuticals™ sunscreen (S1); C – nTiO$_2$ extracted from Boots Soltan™ sunscreen (S2); Di and Dii – nTiO$_2$ extracted from The Body Shop™ liquid foundation (P1). Left panel shows scanning transmission electron microscope image of samples; other panels show false coloured EDS image of element as labelled.

SI.1.2 Powder X-ray diffraction (P-XRD)

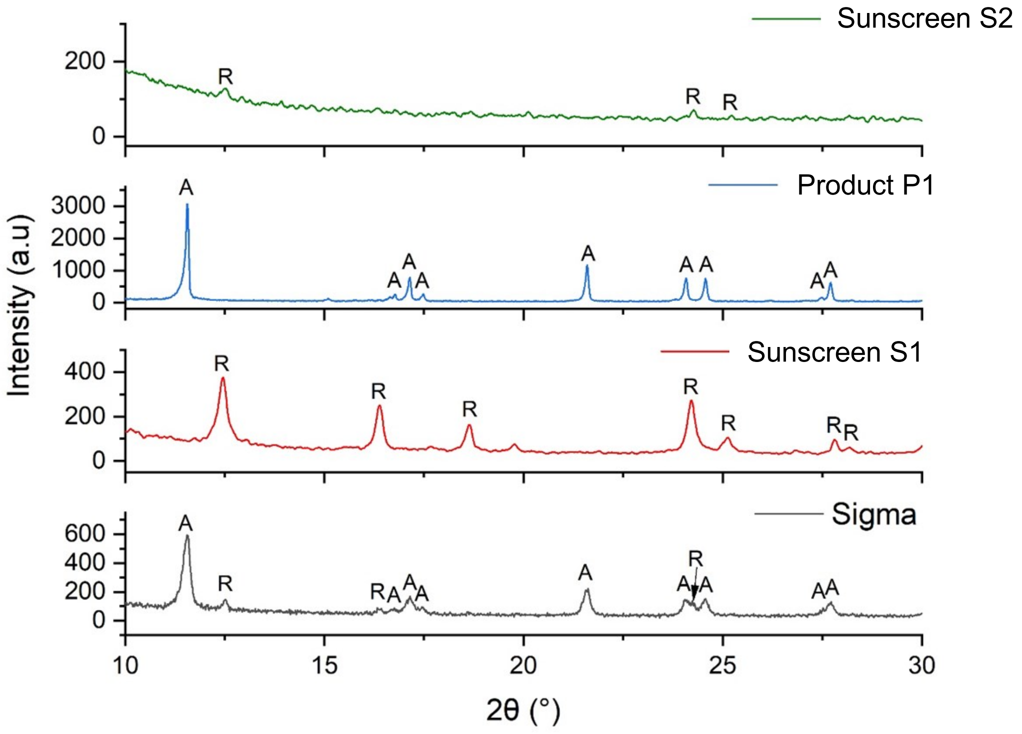

Figure SI.2. Powder X-ray diffraction patterns of samples, with anatase (A) and rutile (R) planes indexed to JCPDS card numbers 21-1272 and 21-1276 for the tetragonal structures of anatase and rutile TiO_2, respectively, as labelled. Peaks at 11.5°, 17.2°, 21.6°, 24.1°, 24.6°, 27.8°, 30.1°, and 30.7° can be indexed to the (101), (004), (200), (105), (211), (118), (116), and (220) planes of the tetragonal anatase crystal system. Major peaks observed at 12.5°, 16.4°, 18.7°, 24.2°, 25.1°, 27.8°, 28.2°, and 30.1° can be indexed to the (110), (101), (111), (211), (220), (002), (310), and (301) planes of rutile TiO_2.

SI.2 Flow cytometric analysis

Flow cytometry was carried out using a Becton Dickinson Fortessa flow cytometer. The laser parameters used during data collection are outlined in Table SI.1.

To monitor cell densities of *Prochlorococcus sp.* MED4 during toxicity testing a 1 mL sub-sample of each replicate was analysed at each time-point. Data was collected three times per replicate to ensure accuracy of the flow cytometric output. For cell-dense cultures grown in nutrient-rich media, samples were diluted 10-fold in autoclaved seawater prior to data collection. Reference beads (2.2 μm high Intensity fluorescent Nile Red particles (Spherotech FH-2056-2)) were added to samples (10 μL) to act as a reference for cell count quantification. Gates set-up using BD FACSDiva software were used to quantify the number of reference beads and free-living cyanobacteria cells and samples were run at a medium flow-rate until 100 reference beads were counted by the software. Subsequently, cell counts were calculated using Microsoft Excel.

Table SI.1 Laser parameters used for flow cytometry.

Parameter	Measure	Log	Voltage (V)
FSC	Area	Yes	600
SSC	Area	Yes	200
B488-530/30	Area	Yes	435
B488-710/50	Area	Yes	456
R640-670/14	Area	Yes	593
R640-730/45	Area	Yes	599
R640-780/60	Area	Yes	418
V405-450/50	Area	Yes	408
V405-525/50	Area	Yes	482
V405-540/30	Area	Yes	569
V405-586/15	Area	Yes	597
V405-610/20	Area	Yes	690
V405-670/30	Area	Yes	606
UV335-450/50	Area	Yes	392
UV355-530/30	Area	Yes	550
YG561-586/15	Area	Yes	455
YG561-610/20	Area	Yes	300
YG561-670/30	Area	Yes	541
YG561-710/50	Area	Yes	435
YG561-780/60	Area	Yes	469

SI.2.1. Total event number obtained during flow cytometric analysis: inferring $nTiO_2$ behaviour

To aid our understanding of $nTiO_2$ behaviour during experiments, NSW was spiked with $nTiO_2$ to make up standards at each concentration (0-100 mg L^{-1}) in the absence of cyanobacteria to assess the extent of $nTiO_2$ aggregation and precipitation during flow cytometry, as control analyses. By sampling these standards as with cultures, it was possible to infer the abundance of $nTiO_2$ aggregates in suspension by recording the total event number (Figure SI.2). Here, it is assumed that events represent suspended $nTiO_2$ aggregates, as all analysed samples were obtained from the midpoint of flasks. In $nTiO_2$ suspensions in the mg L^{-1} range, the number of suspended aggregates reduced greatly within the initial stages of addition to seawater. This is most evident at the 100 mg L^{-1} concentrations where total event number decreased from approximately 37500 to 500 within 24 h. At lower concentrations within the μg L^{-1} range the recorded event number was considerably lower and did not show great variation throughout the course of the experiment. Following 72 h, the number of events appears to stabilise across all concentrations, indicating that despite differing initial concentration, the number $nTiO_2$ aggregates present in suspension did not vary greatly between treatments at later stages of exposure.

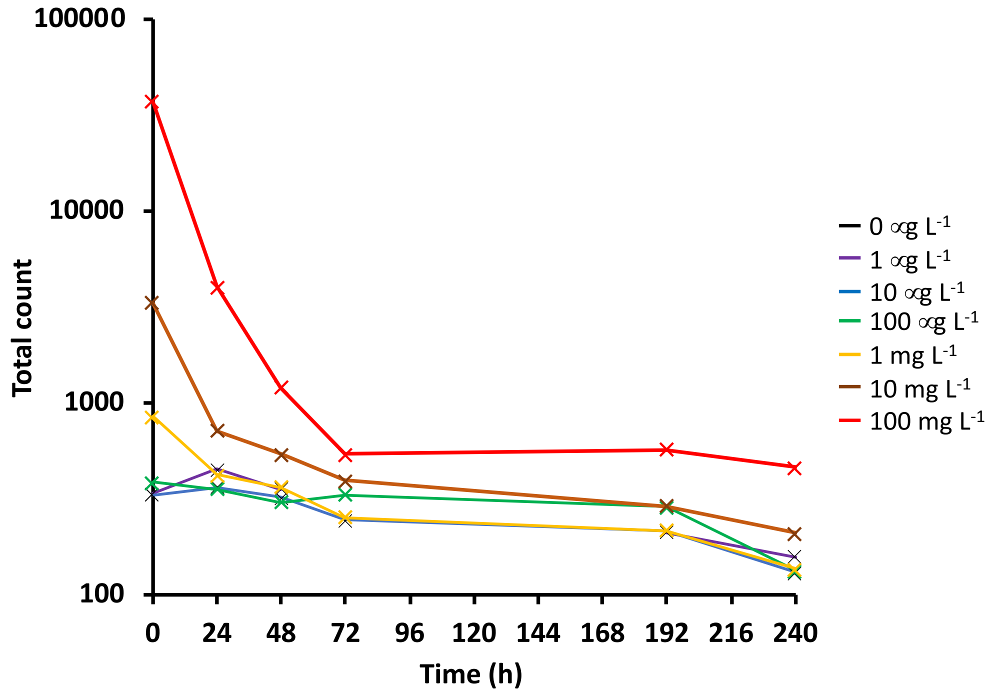

Figure SI.3. Total event count obtained from flow cytometric analysis of cell-free $nTiO_2$ suspensions in natural seawater.

SI.3. DLS data and supporting information

The aggregation behaviour of research-grade $nTiO_2$ (Sigma Aldrich, 19.9±6.6 nm (TEM)) was investigated using dynamic light scattering (DLS). To record precipitation of nanoparticles during the experiment, photographs were captured at regular intervals throughout (see Figure SI.3).

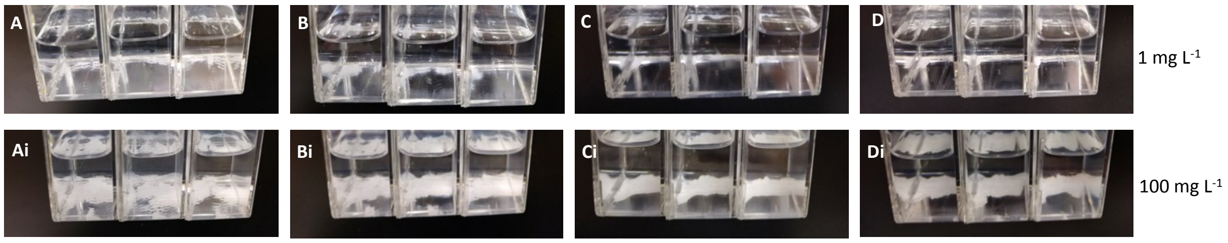

Figure SI.4. Photographic observation of sedimentation of $nTiO_2$ triplicate samples during DLS analysis; A/Ai – 24 h, B/Bi – 168 h, C/Ci – 240 h, D/Di – 336 h.

SI.4 Shotgun Proteomic analysis of short-term $nTiO_2$ exposure

a) Sample collection

After 24 h incubation, samples were immediately placed on ice and centrifuged for 10 minutes at 4 °C at 4000 rpm. The supernatant containing the extracellular proteome was subsequently collected and filter-sterilised using a 0.22 μm filter before being stored at -20 °C. The resultant cell pellet was kept on ice and transferred to a 1.5 mL Eppendorf. Following this the cell pellet was further centrifuged for 1 minute at 13,000 rpm at 4 °C. Supernatant was then discarded and the pellet snap-frozen on dry ice. Pellets were subsequently stored at -20 °C before further processing.

b) TCA protein precipitation of extracellular proteomes

Supernatants were thawed at room temperature and underwent trichloroacetic acid (TCA) protein precipitation as previously described.[1] For 30 mL extracellular proteome samples that had previously been filtered, 750 μL 0.6 % (w/v) sodium deoxycholate (DOC) was added and vortexed to mix. Samples were incubated at room temperature for 10 minutes before adding 1.8 mL 50% trichloroacetic acid (TCA) and incubating on ice for a further 30 minutes. Subsequently, samples were then centrifuged for 15 minutes at 4 °C at 4000 rpm and the supernatant was discarded. The resultant pellet containing exoproteome proteins was resuspended in 750 μL Milli-Q water and transferred to a 1.5 mL Eppendorf. 28 μL 0.6 % DOC was added and the solution was incubated for 10 minutes at room temperature, followed by the addition of 67.5 μL 50 % TCA and incubation on ice for 30 minutes as previous. Eppendorfs were then centrifuged for 10 minutes at 4 °C at 8000 rpm and the supernatant was discarded. Finally, pellets were resuspended in 750 μL ethanol/ether (1:1 v/v) and centrifuged for a further 15 minutes at 4 °C at 13,000 rpm. The supernatant was carefully removed by pipette and the pellet left to dry completely. Once dried, extracellular proteome pellets were stored at -20 °C.

c) Preparation and processing of protein gels

Cell and extracellular proteome pellets were resuspended in 1x LDS buffer (ThermoFisher) containing 1% beta-marcaptoethanol and run on a NuPage 4-12% Bis-Tris precast polyacrylamide gels as previously done.[2] Firstly, cell and extracellular proteome pellets were resuspended in 300 μL and 70 μL LDS beta-mercaptoethanol respectively and vortexed to mix. To ensure samples were well-suspended samples were first placed on a 95 °C hot plate for 5 minutes and subsequently vortexed, this process was repeated three times. Following this samples were sonicated for 5 minutes and heated once again for 5 minutes at 95 °C, this process was also repeated three times. Prior to loading, samples were maintained at 95°C. Samples (30 μL) were subsequently loaded into 10-well NuPage 4-12% Bis-Tris Novex Gels, separate gels for cellular and extracellular samples. Two wells were left blank between control and treated samples to ensure no cross-contamination occurred. The voltage was set at 200 V and gels run for 5 minutes. Following this, gels were washed with Milli-Q water and stained using SafeStain for 30 minutes. Gels were washed following staining three times using Milli-Q water and left overnight. Subsequently, gel bands for proteomic analysis were cut into small

cubes (approx. 2-4 mm) using a sterile blade and placed in 50% ethanol- 50 mM ammonium bicarbonate (ABC) overnight and maintained at 5 °C to aid the de-staining process.

d) In-gel protein digestion

In-gel trypsin digestion and peptide recovery was performed.[3] To remove the stain, gels were washed three times in 50% ethanol 50mM ABC for 20 minutes whilst heated to 55 °C under shaking (650 rpm). Following washing 100% ethanol was added to dehydrate the gel for 5 minutes whilst shaken (650 rpm) at room temperature. Subsequently, 10 mM TCEP 40 mM CAA was added and shaken gently for 5 minutes at 70 °C. The 50% ethanol 50 mM ABC washing procedure was repeated at room temperature and gel samples dehydrated using 100% ethanol, as previous. Following this, gels were hydrated with 2.5 ng L^{-1} Trypsin solution for 10 minutes at room temperature, after which sufficient 50 mM ABC was added to cover gels and samples were left overnight at 37 °C. The following day peptides were extracted by sonication (5-10 minutes) with the addition of 25% acetonitrile 5% formic acid. Following sonication, all liquid extractions were transferred to a clean 1.5 mL Eppendorf. This process was repeated three times. Extracted peptides were concentrated using a Speed-vac set at 45 °C and resuspended in 50 μL 2% CAN 0.1% TFA. To ensure no gel passed through to the final sample for analysis, all resuspended samples were filtered using a 0.22 μm Costar® Spin-X® centrifuge tube before being stored at -20 °C.

SI.4.1 Proteomics results:

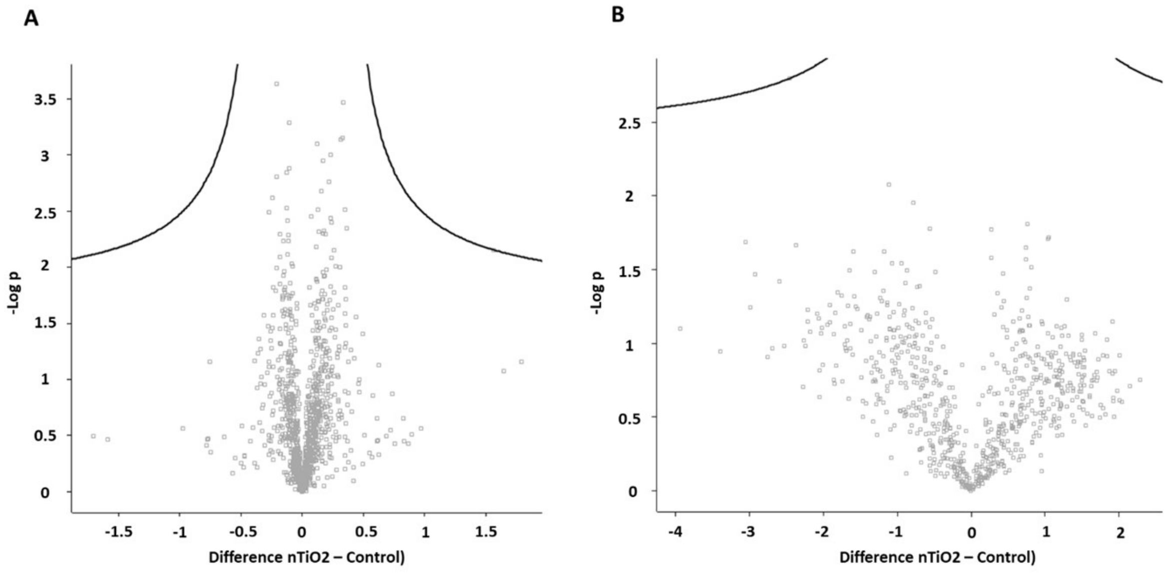

Figure SI.5. Volcano plot of cellular (A) and extracellular (B) proteomes.

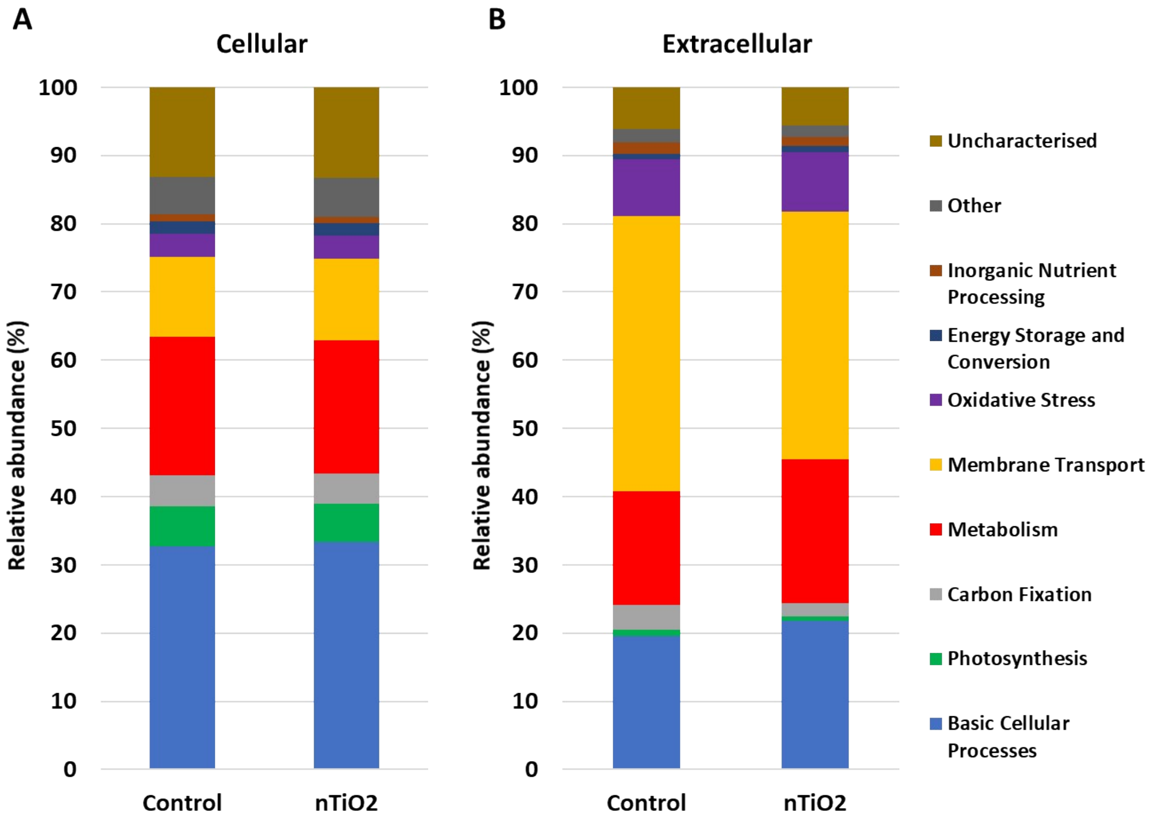

Figure SI.6. Relative abundance of protein groups, classified in terms of biological function as listed on the Uniprot database and KEGG assignment, identified by proteomic analysis of; A) cellular and B) extracellular samples of *Prochlorococcus sp.* MED4 exposed to research-grade $nTiO_2$ (100 $\mu g\ L^{-1}$) for a period of 24 h. Data is presented as the mean of three biological replicates.

SI.5 Investigating the effect of consumer $nTiO_2$ upon natural marine communities.

SI.5.1 16S/18S rRNA gene amplicon sequencing: Additional information

a) Library Preparation

The 16S rRNA gene v4-5 regions (515F-Y and 926R primers) and 18S rRNA gene v8-9 regions (V8F and 1510R primers) were amplified using the Q5® Hot Start High-Fidelity 2X Master Mix (New England Biolabs® inc.). PCR conditions were as follows; 16S rRNA: 98°C (30 s), 25 cycles of: 98°C (10 s), 50°C (15 s), 72°C (20 s), 72°C (5 min), and cool to 4°C; 18S rRNA: 98°C (3 min), 25 cycles of: 98°C (20 s), 65°C (15 s), 72°C (15 s), and cool to 4°C. Following PCR, Ampliclean magnetic beads (Nimagen) were used to purify the PCR product. Normalisation was carried out using a SequelPrep® Normalisation Plate Kit (ThermoFisher Scientific). NEBNext Library Quant Kit for Illumina (New England Biolabs, UK) was used to pool and quantify the 16S rRNA and 18S rRNA libraries before diluting to 4 nM. Subsequently, 0.2N NaOH was used to denature the libraries and the MiSeq Reagent Kit v3 (600 cycles; Illumina) was used for amplicon sequencing following the manufacturer's instructions for a library of 14 pM, using phiX (2%) and an internal reference. Amplicon sequencing was carried out on an Illumina MiSeq and reads subsequently demultiplexed using BaseSpace (Illumina).

b) *DADA2 Analysis*

The DADA2 pipeline (version 1.8.0) was used to analyse data obtained from sequencing.[4-5] This pipeline was chosen due to its enhanced resolution upon taxonomic assignment compared to other methods.[6] The output from the DADA2 pipeline are referred to as amplicon sequencing variants (ASVs). Here, the SILVA reference database (v132) was used in order to classify ASVs.[7] Prior to downstream analysis, mammalia, chloroplasts, and mitochondria were removed from both the bacterial (16S rRNA) and eukaryotic (18S rRNA) datasets, as well as removing any eukaryotes from the 16S rRNA data and any bacteria or archaea from the 18S rRNA data. On average, 16S rRNA and 18S rRNA data contained an average of 11263 and 28699 reads per sample respectively. Any samples that contained less than 1000 reads were removed from downstream analyses.

c) *Analysis of 16S rRNA and 18S rRNA data using MicrobiomeAnalyst*

16S rRNA and 18S rRNA datasets were analysed using the web-based tool MicrobiomeAnalyst.[8-9] This tool was selected based on its ease-of-use and effectiveness to gain quick insight into possible alterations in alpha- and beta- diversity of microbiome data. Briefly, ASV data was uploaded to the MicrobiomeAnalyst platform and filtered as follows; for low count ASVs, those with less than 2 counts or in the lowest 10% prevalence were removed, as well as those in the lowest 10% based on interquartile range. This resulted in a total of 1316 ASVs being taken forward for analysis in the 16S rRNA dataset, and 1480 ASVs in the 18S rRNA dataset respectively. No rarefication was applied as read counts did not vary substantially between samples in either the 16S or 18S rRNA datasets. Total sum scaling was performed on data and no transformation was applied. Subsequently, various measures of alpha diversity were calculated to assess differences in species richness and evenness between treatments. Relative abundance of major bacterial (16S rRNA dataset) and eukaryotic (18S rRNA dataset) phyla were then calculated and bar charts produced to visualise this data. To assess any statistically significant differences between communities belonging to individual treatments, PCoA was carried out (PERMANOVA based on Bray-Curtis dissimilarity) were used to assess differences in the 16S rRNA and 18S rRNA datasets respectively. Figures for both relative abundance and PCoA plots were downloaded and utilised to present data. Relative abundance values were utilised for subsequent statistical analysis via two-way T-tests between control and treated samples and a variety of taxonomic levels.

SI.5.2 16S/18S rRNA amplicon sequencing results

a) *Alpha diversity*

Table SI.2. Alpha diversity of bacterial communities

			Species Richness		Species Evenness	
**Sample**	**No. of replicates**	**No. of ASVs**	**Chao 1**	**ACE**	**Shannon**	**Simpson**
Control	3	751 ± 44	825.55 ± 54.32	864.11 ± 47.25	4.10 ± 0.15	0.89
Extracted $nTiO_2$ (S2)	5	758 ± 46	837.85 ± 51.89	878.12 ± 52.89	4.01 ± 0.30	0.88
Neat Sunscreen (S2)	3	586 ± 174	655.79 ± 172.37	677.21 ± 161.69	4.06 ± 0.10	0.91

Table SI.3. Alpha diversity of eukaryotic communities

			Species Richness		Species Evenness	
**Sample**	**No. of replicates**	**No. of ASVs**	**Chao 1**	**ACE**	**Shannon**	**Simpson**
Control	3	990 ± 73	1052.71 ± 60.62	1062.45 ± 61.75	4.74 ± 0.93	0.93
Extracted $nTiO_2$ (S2)	5	926 ± 80	989.40 ± 74.72	1005.83 ± 67.96	5.21 ± 0.08	0.98
Neat Sunscreen (S2)	4	767 ± 180	823.66 ± 175.81	837.70 ± 169.45	4.92 ± 0.39	0.97

b) Community composition

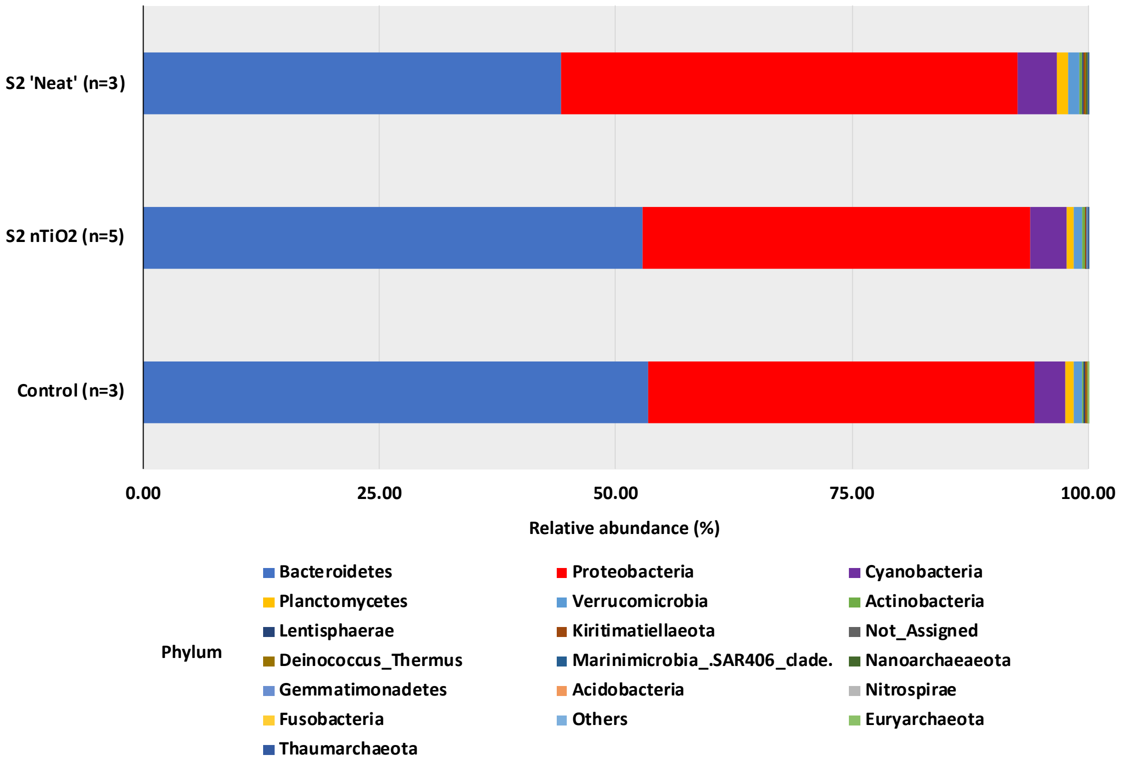

Figure SI.7. Relative abundance of major prokaryotic phyla in each treatment as identified by 16S rRNA amplicon sequencing

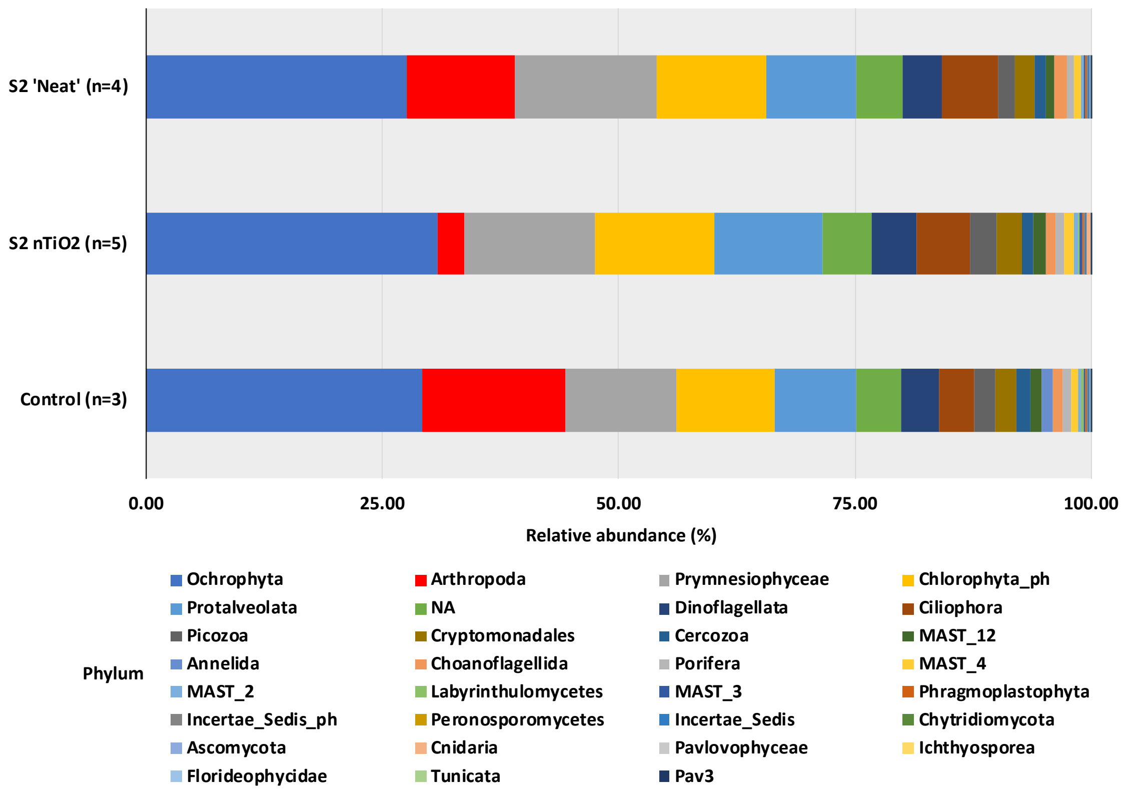

Figure SI.8. Relative abundance of major eukaryotic phyla in each treatment as identified by 18S rRNA amplicon sequencing

References

1. Christie-Oleza, J. A.; Armengaud, J., In-depth analysis of exoproteomes from marine bacteria by shotgun liquid chromatography-tandem mass spectrometry: the Ruegeria pomeroyi DSS-3 case-study. *Mar Drugs* **2010,** *8* (8), 2223-39.
2. Zadjelovic, V.; Chhun, A.; Quareshy, M.; Silvano, E.; Hernandez-Fernaud, J. R.; Aguilo-Ferretjans, M. M.; Bosch, R.; Dorador, C.; Gibson, M. I.; Christie-Oleza, J. A., Beyond oil degradation: enzymatic potential of Alcanivorax to degrade natural and synthetic polyesters. *Environ Microbiol* **2020,** *22* (4), 1356-1369.
3. Shevchenko, A.; Tomas, H.; Havlis, J.; Olsen, J. V.; Mann, M., In-gel digestion for mass spectrometric characterization of proteins and proteomes. *Nat Protoc* **2006,** *1* (6), 2856-60.
4. Callahan, B. J.; McMurdie, P. J.; Rosen, M. J.; Han, A. W.; Johnson, A. J.; Holmes, S. P., DADA2: High-resolution sample inference from Illumina amplicon data. *Nat Methods* **2016,** *13* (7), 581-3.
5. Callahan, B. J.; Sankaran, K.; Fukuyama, J. A.; McMurdie, P. J.; Holmes, S. P., Bioconductor Workflow for Microbiome Data Analysis: from raw reads to community analyses. *F1000Res* **2016,** *5*, 1492.
6. Hugerth, L. W.; Andersson, A. F., Analysing Microbial Community Composition through Amplicon Sequencing: From Sampling to Hypothesis Testing. *Front Microbiol* **2017,** *8*, 1561.
7. Quast, C.; Pruesse, E.; Yilmaz, P.; Gerken, J.; Schweer, T.; Yarza, P.; Peplies, J.; Glockner, F. O., The SILVA ribosomal RNA gene database project: improved data processing and web-based tools. *Nucleic Acids Res* **2013,** *41* (Database issue), D590-6.
8. Chong, J.; Liu, P.; Zhou, G.; Xia, J., Using MicrobiomeAnalyst for comprehensive statistical, functional, and meta-analysis of microbiome data. *Nat Protoc* **2020,** *15* (3), 799-821.
9. Dhariwal, A.; Chong, J.; Habib, S.; King, I. L.; Agellon, L. B.; Xia, J., MicrobiomeAnalyst: a web-based tool for comprehensive statistical, visual and meta-analysis of microbiome data. *Nucleic Acids Res* **2017,** *45* (W1), W180-W188.
