# Supplementary figures and images for: Environmentally relevant concentrations of titanium dioxide nanoparticles pose negligible risk to marine microbes
Source: Environ Sci Nano. 2021 Apr 9;8(5):1236–55. doi: 10.1039/d0en00883d (PMC8136324; doi:10.1039/d0en00883d)

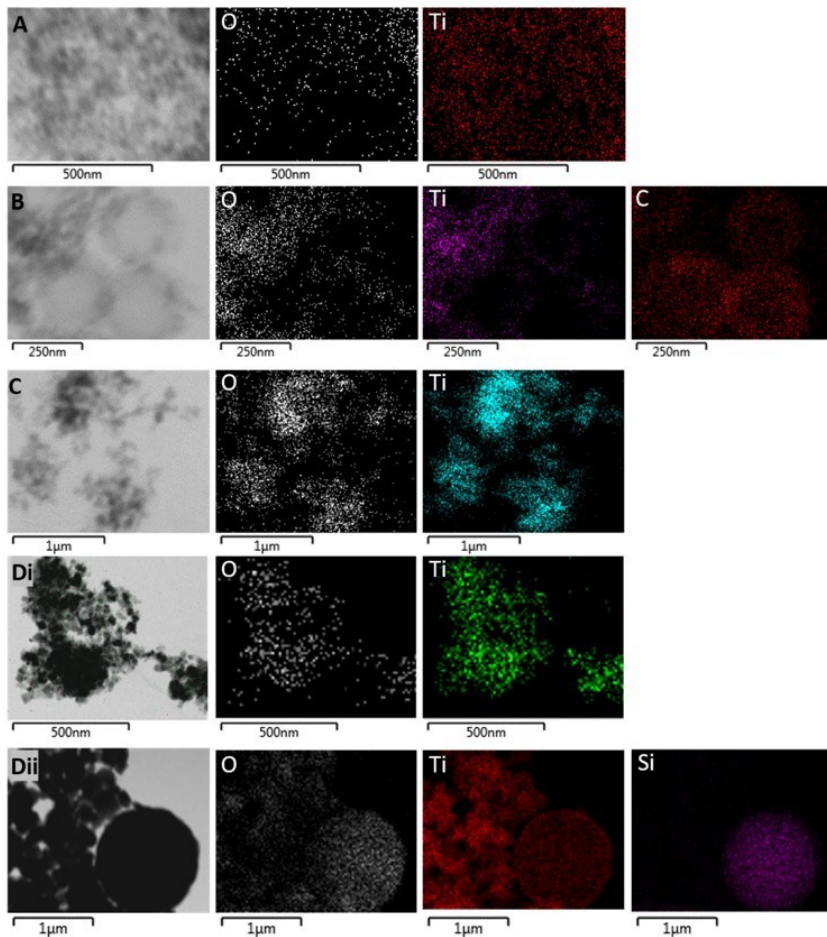

Supplement: EN-008-D0EN00883D-s005 [file EN-008-D0EN00883D-s005.pdf]

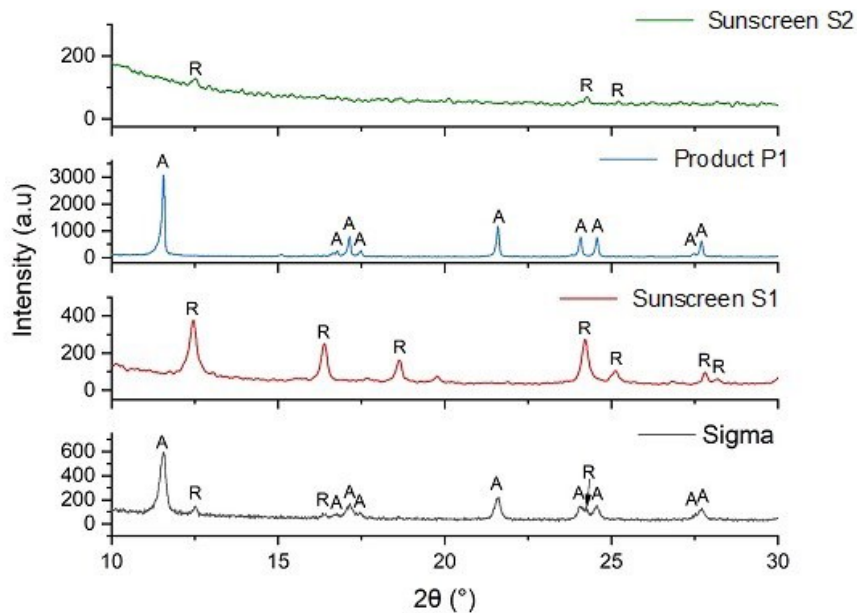

Supplement: EN-008-D0EN00883D-s006 [file EN-008-D0EN00883D-s006.pdf]

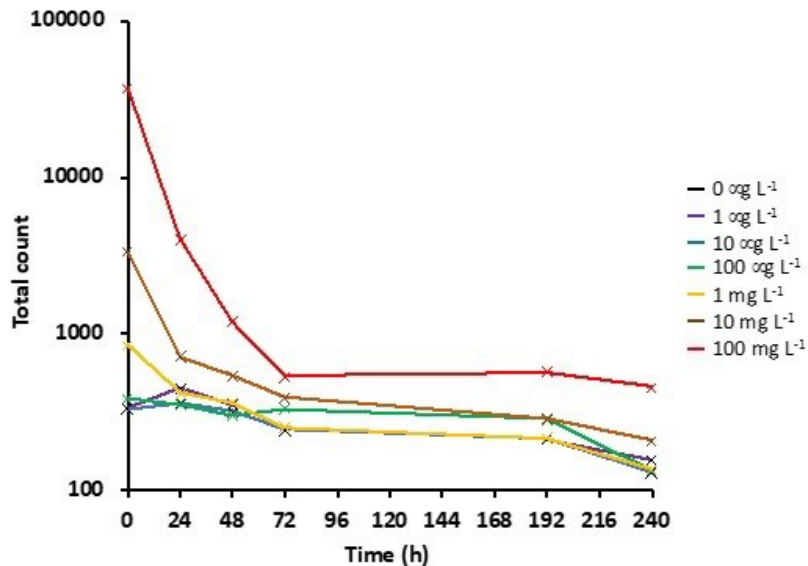

Supplement: EN-008-D0EN00883D-s007 [file EN-008-D0EN00883D-s007.pdf]

**A**

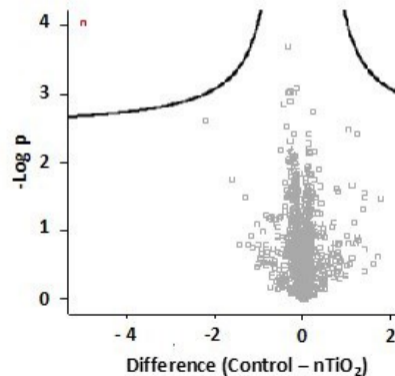

**B**

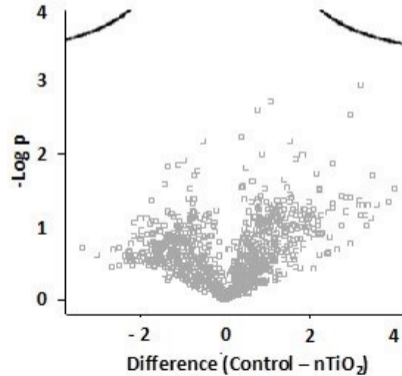

Supplement: EN-008-D0EN00883D-s009 [file EN-008-D0EN00883D-s009.pdf]

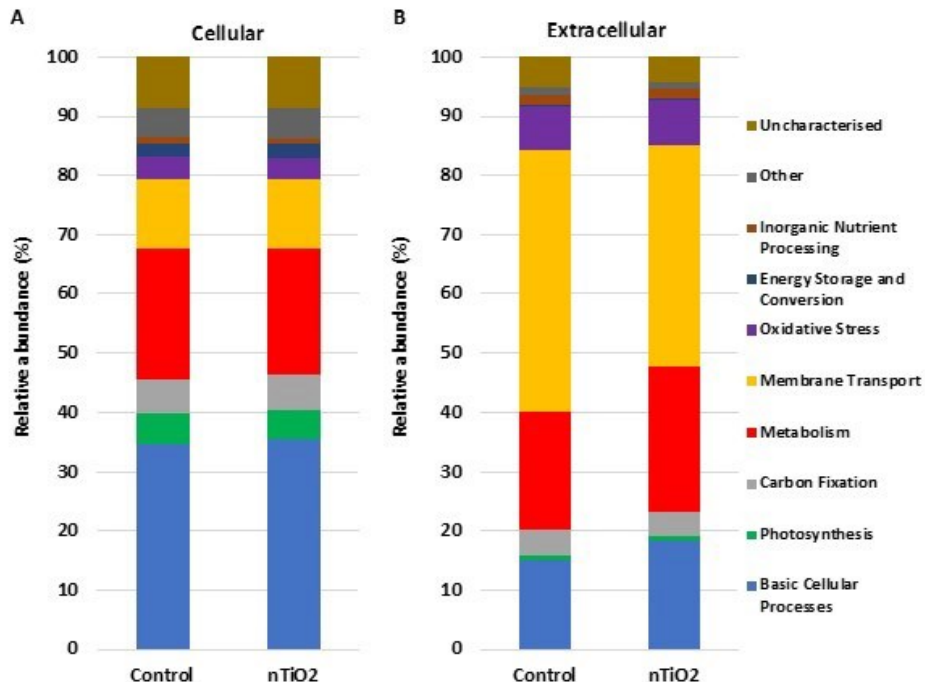

Supplement: EN-008-D0EN00883D-s010 [file EN-008-D0EN00883D-s010.pdf]

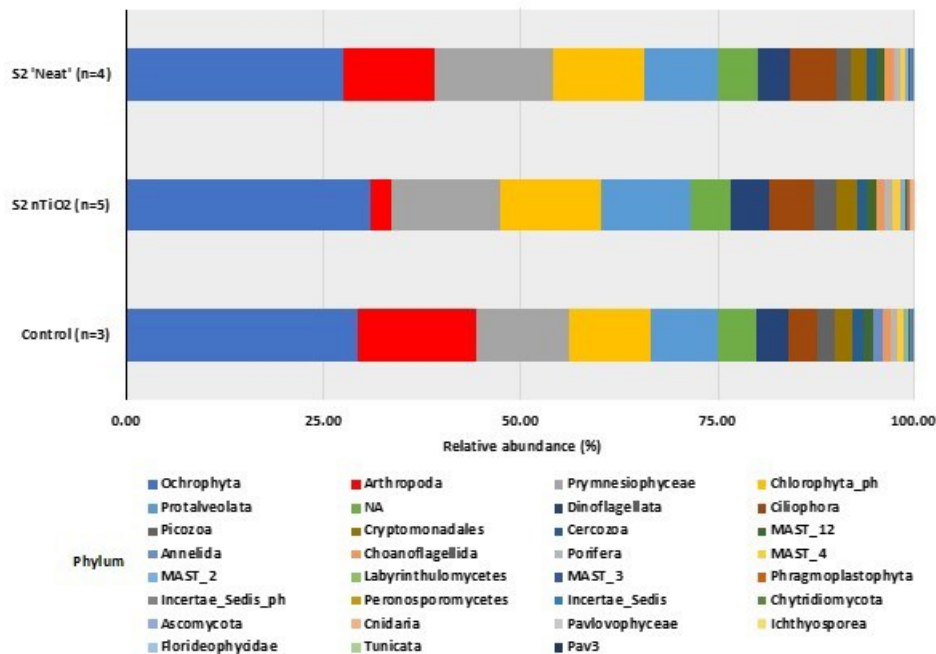

Supplement: EN-008-D0EN00883D-s012 [file EN-008-D0EN00883D-s012.pdf]
