# Supplementary material for: Environmentally relevant concentrations of titanium dioxide nanoparticles pose negligible risk to marine microbes
Source: Environ Sci Nano. 2021 Apr 9;8(5):1236–55. doi: 10.1039/d0en00883d (PMC8136324; doi:10.1039/d0en00883d)

Electronic Supplementary Material (ESI) for Environmental Science: Nano.
This journal is © The Royal Society of Chemistry 2021

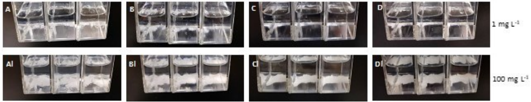

Supplement: EN-008-D0EN00883D-s008 [file EN-008-D0EN00883D-s008.pdf]
